# Deciphering the immune microenvironment of a tissue by digital imaging and cognition network

**DOI:** 10.1038/s41598-018-34731-x

**Published:** 2018-11-12

**Authors:** A. Lopès, Al H. Cassé, E. Billard, E. Boulcourt-Sambou, G. Roche, C. Larois, N. Barnich, S. Naimi, M. Bonnet, B. Dumas

**Affiliations:** 1 Clermont Université, UMR 1071 Inserm/Université Clermont-Auvergne, 63000 Clermont-Ferrand, France; 2 INRA, USC-2018, 63000 Clermont-Ferrand, France; 3 Research Biologics, Sanofi R&D, 94400 Vitry-Sur-Seine, France; 4 Histopathology and Bio-Imaging Group, Sanofi R&D, 94400 Vitry-Sur-Seine, France

## Abstract

Evidence has highlighted the importance of immune cells in various gut disorders. Both the quantification and localization of these cells are essential to the understanding of the complex mechanisms implicated in these pathologies. Even if quantification can be assessed (e.g., by flow cytometry), simultaneous cell localization and quantification of whole tissues remains technically challenging. Here, we describe the use of a computer learning-based algorithm created in the Tissue Studio interface that allows for a semi-automated, robust and rapid quantitative analysis of immunofluorescence staining on whole colon sections according to their distribution in different tissue areas. Indeed, this algorithm was validated to characterize gut immune microenvironment. Its application to the preclinical colon cancer APC^Min/+^ mouse model is illustrated by the simultaneous counting of total leucocytes and T cell subpopulations, in the colonic mucosa, lymphoid follicles and tumors. Moreover, we quantify T cells in lymphoid follicles for which quantification is not possible with classical methods. Thus, this algorithm is a new and robust preclinical research tool, for investigating immune contexture exemplified by T cells but it is also applicable to other immune cells such as other myeloid and lymphoid populations or other cellular phenomenon along mouse gut.

## Introduction

An increasing number of studies have shown the importance of monitoring mucosal immune responses in gut disorders. An exacerbated immune response is the hallmark of inflammatory bowel diseases^1–4^, whereas an impaired immune response is known to be associated with poor prognosis or progression of colorectal cancer (CRC)^5–8^. Because the involved mechanisms remain unclear, novel approaches to study the gut immune system are needed.

Several methods were developed to characterize, quantify and/or localize immune cells in gut tissues. Flow cytometry (FC) is commonly used because it allows simultaneous cellular and functional analysis^9–12^. However, FC does not allow precise immune cell localization or cell-cell interaction characterization. Furthermore, preparing viable single cell suspensions from solid tissues for FC analysis remains challenging and limits the number of sample. Large tissue sections are necessary to reliably monitor the number of interesting cells, which prevents the study of small key areas, such as preneoplastic lesions in oncology. Another approach is cell detection and localization via immunohistochemistry (IHC) with either global manual scoring or quantification on a limited tissue area. However, manual analysis is laborious, time consuming and inaccurate. Indeed, quantification is performed on a few random fields chosen by the experimenter^13^. More recently, a new method called “Imaging Mass Cytometry” (IMC) enables efficient immune cell quantification and localization on mouse liver slides^14^. However, IMC complex data analysis is both time-consuming and expensive and requires sophisticated software and high expertise in bioinformatics^15,16^.

In recent decades, the development of digital image analysis (DIA) has provided alternative solutions for tissue section analysis through rapid and automated segmentation of immunostained cells. A batch of images can be automatically analysed using a customized algorithm^13,17^. An important use of these methods in gut pathologies is probably the Immunoscore^6^, a new prognostic tool for CRC patients that uses *in situ* quantification of CD3^+^ tumor-infiltrating T cells^18^. DIA was also used in murine colitis models to study the gut microenvironment, as reported on whole mouse colon sections, via an automated method to evaluate inflammatory areas^19^, but DIA was not used for single cell analysis. Moreover, there is no general method to obtain the fine localization and number of immune cells along a mouse colon.

Here, we present a DIA semi-automated process using Tissue Studio software (version 2.6) to simultaneously discriminate, localize and quantify immune cell populations within distinct areas of a whole colon section. We chose the APC^Min/+^ mouse model to validate this approach, as it is a preclinical reference model for CRC^20,21^. Three areas were targeted: the colonic mucosa, lymphoid follicles and tumors, focusing on T cells, the significance of which is well-established in CRC^5–7,9,18,22,23^. We validated the precision and robustness of our method on several colon slides and with different fluorescence patterns despite the tissue feature complexity and background. This pipeline allows precise characterization of the immune microenvironment in small tissue structures, such as lymphoid follicles, and was built to be used to other intestinal tissues and other gut diseases.

## Results

### General DIA procedure based on Cognition Network technology (CNT)

Immunostaining was performed using specific membrane fluorescent labelling associated with Tyramide Signal Amplification, as previously described^6,24–27^ (See Supplementary Fig. S1). This staining was completed with DAPI nuclear counterstaining for cell identification and numbering within tissues. We chose paraffin-embedding to preserve the colon structure and allow optimal sample quality for slide digitalization and image analysis processing. Moreover, the fluorescence signal to noise ratio must be managed to avoid signal saturation and allow accurate tissue and cell delimitation.

**Figure 1:**
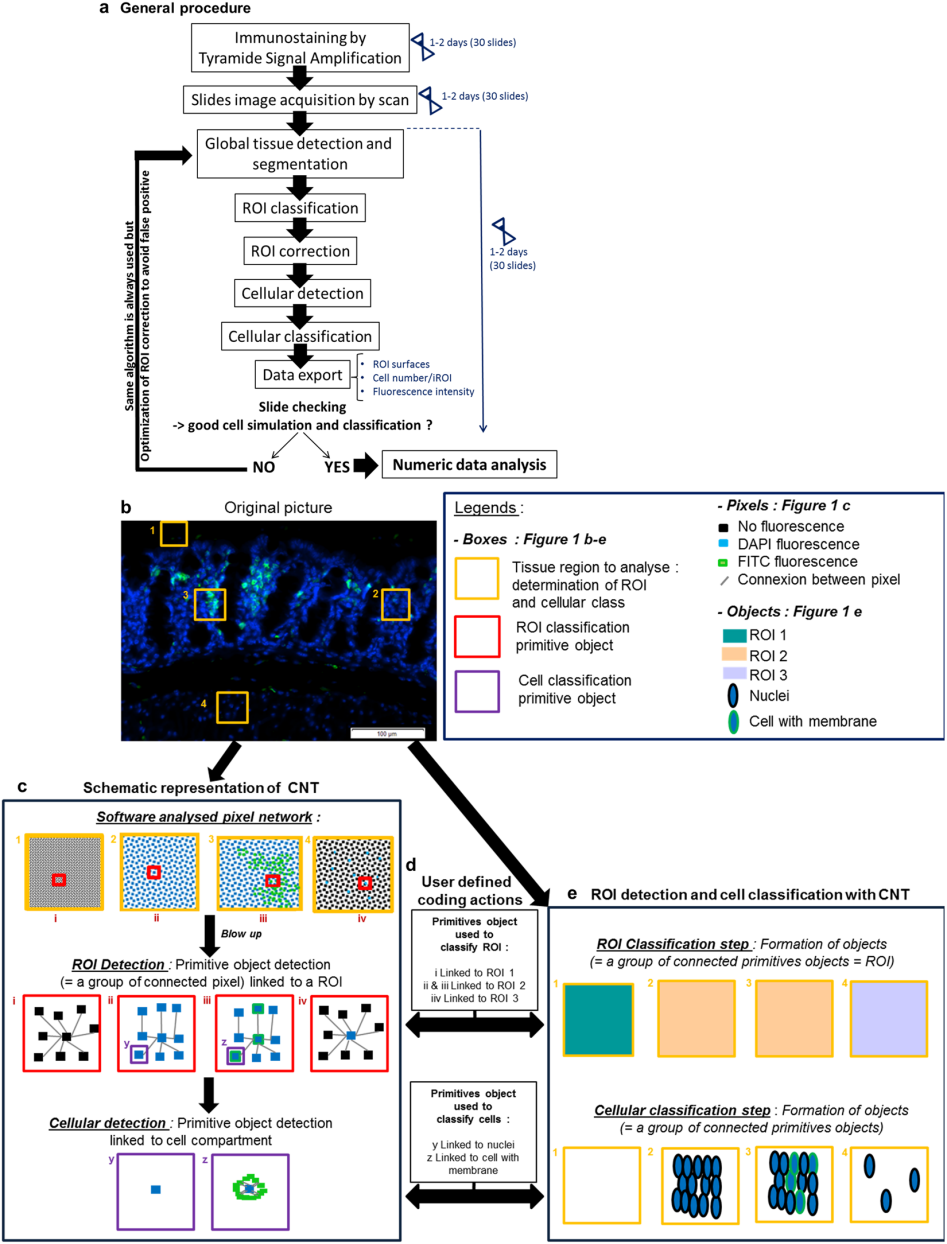
Immune cell quantification general procedure and Cognition Network Technology (CNT) principle. (**a**) The procedure to quantify immune cells is based on a computer learning algorithm and elaborated using Tissue Studio software (Definiens, Munich, Germany). After optimal image acquisition, we evolved the algorithm using five sequential steps: global tissue detection, ROI (Region Of Interest) classification, ROI correction to discriminate the ROIs associated with APC^Min/+^ colon tissue, cellular detection and cellular classification to quantify positive membrane stained cells per ROI. (**b**–**e**) Particular blow up of a colon region is shown as an example of the digital image analysis process. Information is extracted from the picture using a hierarchy of image-objects, formed by grouping of pixels, after an evolutionary alternation of classification and segmentation steps. For example, in blow up picture (**b**), areas (yellow boxes) are selected to distinguish various ROIs (ROI 1: box 1; ROI 2: box 2 and 3; and ROI 3: box 4). These selected zones are recorded as a pixel network with CNT (**c**, top boxes). The image is analysed at the pixel level in a contextual fashion. Specific groups of connected pixels, named “primitive objects” are identified and shown in **c** (middle red boxes). Then each primitive object is linked with a specific ROI by a coded action (**d**, top box; ex: primitive object (I) is linked with ROI 1). The same connected primitive objects are finally grouped to form an object associated to a specific ROI as a result of the recognition of ROI-associated primitive-objects. This permits the classification of each selected colon zones into respective ROI (**e**, top boxes; ROI 1: Green; ROI 2: Yellow; ROI 3: Blue). The same process is applied for cellular analysis (**c**, bottom purple boxes), with a more segmented image reaching cellular scale. An object is defined as a cell (**e**, second line). In that case, primitive objects (**c**, bottom purple boxes) are smaller, and linked to a cell type (**d**, second line).

To decipher the immune microenvironment in distinct regions of interest (ROIs) in the APC^Min/+^ colon, we had to develop the optimised DIA algorithm with Tissue Studio software (Definiens, Germany). It was not possible to use the pre-calibrated algorithm available in the software, because of the complexity of detecting multiple cell types in different whole colon regions with precise cell numbering for each ROI. But, the friendly interface of the software allows users to build their new specific algorithm adapted to their scientific problematic and sample. More precisely, users can select, calibrate and organize an extensive series of rules to analyse their images, which are later translated by software in comprehensive language, to allow the automatic execution of the algorithm by the computer. Thus, we have optimised a new algorithm for a whole colon section DIA, by selecting, configuring and classifying various Tissue Studio software functions and rules. The global structure of the developed algorithm is presented in Fig. 1a. This set of commands was developed using an exhaustive catalogue of pre-coded functions, which need to be set and ordered. A brief summary of possible functions and its associated non-configured parameters are described in Supplementary Table 1 together with the available ruleset combination numbering.

All of the rulesets were based on Definiens Cognition Network Technology (CNT)^28^, an object-based image analysis method, which extracts information from the image using a hierarchy of pixel group, called “objects” (See Fig. 1b,c). Contrary to classical image analysis, CNT considers pixels not as an isolated entity but in the image frameworks. In practice, it uses the color, the shape, the size, the context, and their relationship with each group of pixels, or object, to analyse images.

These pixel properties were essential to apply this technology to the whole colon slide (See Fig. 1b–e). In the first step of the algorithm, fluorescence from a colon section (exemplified in Fig. 1b) was detected using DAPI and FITC fluorescent channels and segmented into pixel clustering groups called “primitive objects”, as shown in Fig. 1c. The “primitive objects”, which have a specific fluorescence pattern and spatial network, when grouped together, form an “object”. Depending on the algorithm step, we have linked these specific patterns with a respective group of large “objects” called “ROI” (see Fig. 1d, first line: example of 3 classes of ROI in picture; “ROI1”, “ROI2” and “ROI3”) or with a respective group of small “objects” called “cells” (Fig. 1d; second line; example of two classes of cells: “nuclei” and “cell with membrane”). This established link thanks to selection, calibration and ordering of pre-coded rules in Tissue Studio catalogue, allowed the user to distinguish ROIs and cells on the colon section, as described in Fig. 1e. More precisely, we implemented different ROIs corresponding to each colonic structure and classified them using an automatic ROI classification step and manual refining as necessary, as shown in Fig. 1a. Up to 8 ROI classes can be defined with the software. Finally, we quantified immune cells by implementing cellular detection and classification based on the same CNT process using segmentation and classification iterations^28^. We exported various parameters, such as the ROI area, mean fluorescence intensities and number of cells per ROI, for statistical comparison.

### Specific workflow for mouse colon immune cell detection

**Figure 2:**
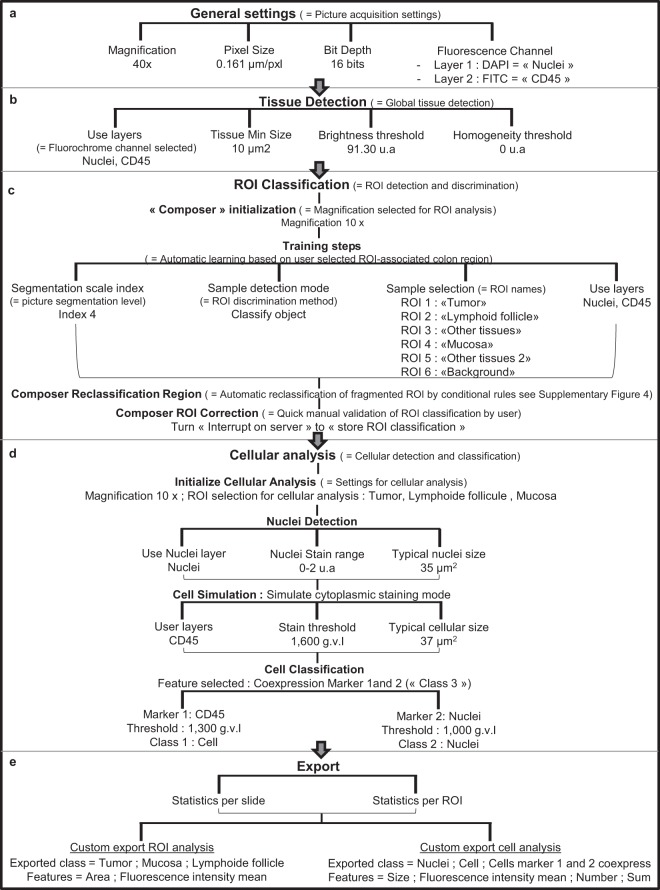
Optimal digital image analysis algorithm flowchart for whole colon slide: example of CD45 single staining. All algorithm functions were chosen and ordered from the exhaustive Tissue Studio software pre-written rule list. They constituted an innovative program that did not exist in software library, and were optimised to quantify cells in various ROIs from a whole colon stained section. It was developed by an iterative method. (**a**) First step, “General settings”, is a fixed step by the software. It allows to set the analysis workflow by indicating all the image file parameters (magnification, resolution and fluorescence staining). (**b**) We implemented a second step, called “Tissue detection” to detect the complete colon tissue on the slide. It is based on user defined optimal parameter settings: fluorescence channel, minimal tissue detected size, and fluorescence brightness and homogeneity. (**c**) We implemented a third step, “ROI Classification”, to discriminate the various colon regions of interest (ROI). To manage with the complexity of a whole organ slide, we implemented three analysis steps, named composers. First composer is “Initialization”, a mandatory composer in Tissue Studio software to detect ROI. It allows automatic recognition of ROIs by software, using a stock of representative areas, selected on colon section during algorithm development. Second composer is “Reclassification region” based on a specific series of conditional rules to automatically reclassify the ROIs which were incorrectly classified by the initialization composer. We have added a third composer, “ROI correction” to allow a quick visual checking of “ROI classification” by user. (**d**) Then, to quantify cells of interest in each ROI, we implemented the “Cellular analysis” step. The first action is a mandatory “Initialize Cellular analysis” function, to set the optimal parameters of cellular analysis (magnification and chosen ROIs). Then, we have chosen and ordered three optimal coded functions and their settings to detect all cells and cells of interest (e.g. CD45^+^ cells here) in all ROIs: Nuclei Detection, Cell Simulation and Cell classification. (**e**) To finalize this algorithm we selected the export command parameters.

The detailed optimised workflow of this new algorithm is described step-by-step, for single (Fig. 2) and double membrane staining (Supplementary Fig. S2). More precisely, CD45^+^ leucocytes were detected using specific FITC labelled antibodies, meanwhile CD4^+^ T cells were detected with CD3 and CD4 membrane proteins using Cy3 and FITC labelled antibodies respectively. In each case, nuclei were stained using DAPI fluorochrome. The stainings were performed on colonic sections scanned at 40x magnification, 0.161028 µm/pixel resolution and 16 bit depth. These picture acquisition parameters fix the first step of the algorithm (See Fig. 2a, “General setting” step”). The other analysis steps such as tissue detection, ROI classification, cellular analysis and export were chosen, organized and calibrated via an iterative method (Fig. 2 and Supplementary Fig. S2). All of these are described in detail below.

### ROI detection and classification for CD45 immunostaining

**Figure 3:**
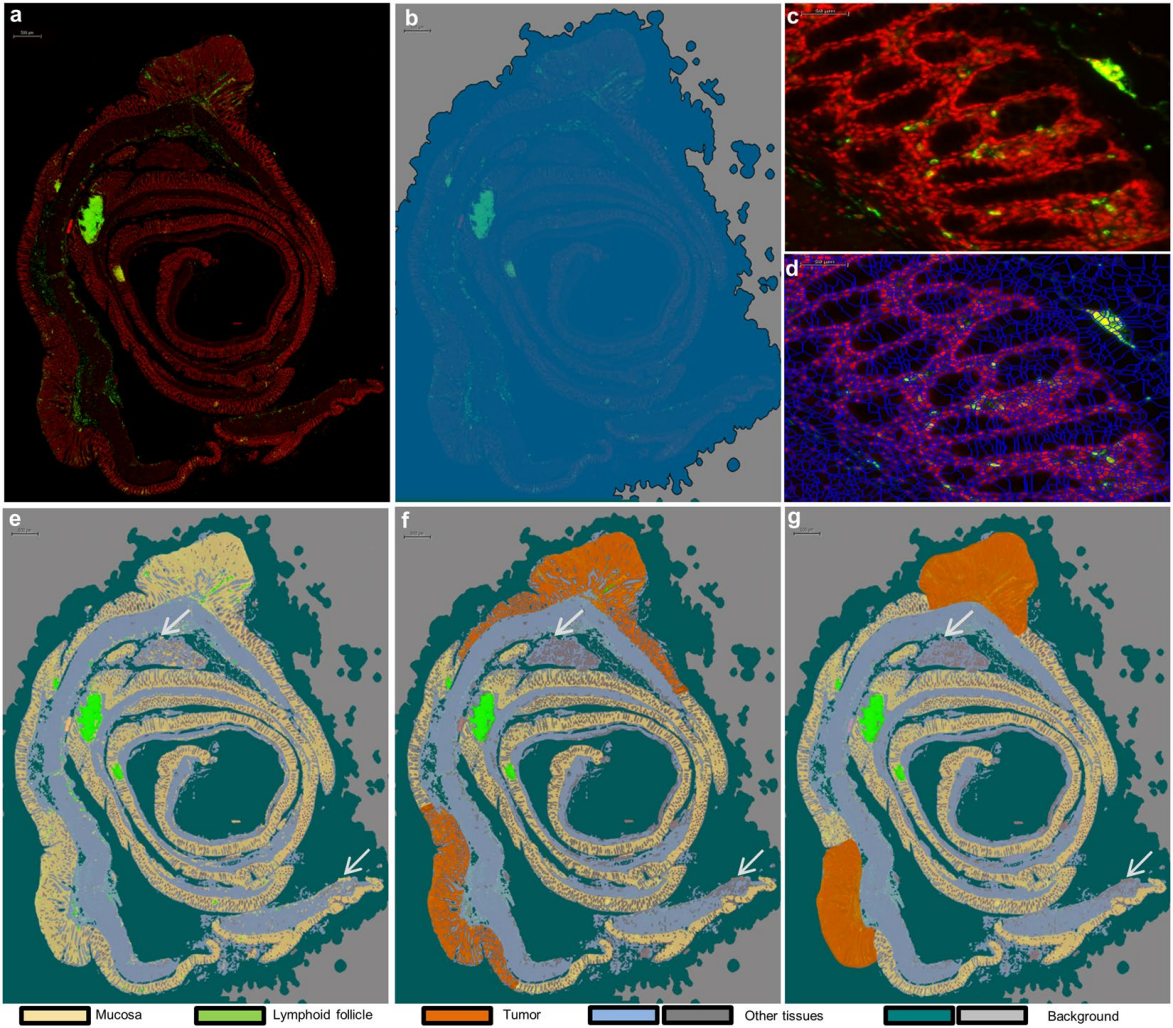
ROI classification method after CD45 immunostaining. (**a**) Representative image of CD45^+^ leucocyte staining (green). We detected the whole colon section using a global tissue detection step and DAPI (red) counterstaining, and (**b)** we simulated data using detected (dark blue) and not detected (light grey) areas. (**c**,**d**) Representative validation of the optimal tissue segmentation index on mucosa with immunostaining (**c**) and segmented mucosa (**d**). (**e**–**g**) For ROI classification, we approved five ROI classes to differentiate between mucosa (beige), lymphoid follicles (green), tumors (orange), other tissues (light blue and dark grey) and background areas (blue green and light grey). (**e**) We first performed automated ROI classification based on the nuclear and membrane fluorescence signals. This step permitted the precise distinction between mucosa (beige), lymphoid follicles (green) and background (blue-green and dark-grey). (**f**) We evolved the automated ROI reclassification step by adding conditional rules to detect tumor (orange) and avoid false mucosa detection (grey area). (**g**) To optimise and control ROI classification before cellular analysis, we added a last step using manual correction. We presented an example of a false ROI classification (white arrow), which was detected as mucosa (**e**). We corrected this error after ROI reclassification as other tissue (dark grey) (**f**,**g**).

To automatically detect the three immune ROIs (iROIs) in the APC^Min/+^ colon: mucosa, lymphoid follicles and tumors (See Supplementary Fig. S3) we first defined the total area to analyse, via automatic global tissue detection using membrane (Fig. 3b) and nuclear stainings (represented in Fig. 3a with alternate red staining). As shown by the flowchart in Fig. 2b, we determined the optimal thresholds for DAPI fluorescence brightness and homogeneity for all slides by up to 10 iterations, on the basis of unstained area exclusion and unique tissue stain area detection at the end of the simulation. Small non-tissue stained areas (<10 µm^2^) were removed from the analysis by adding a minimal size rule. We thus successfully detected a complete colon section (See Fig. 3a: original data; 3b: colon tissue detection by the machine).

Figure 3d shows the result of the segmentation step of the original mucosa image, which is displayed in Fig. 3c. It consists of the fragmentation of detected tissues into “primitive objects”. Cell individualization and ROI classification are based on this second step. A higher segmentation scale creates small “primitive objects”, which facilitates cell individualization. However, it complicates ROI classification, making it impossible to determine a global fluorescence pattern for each ROI. On the contrary, a low segmentation scale allows precise ROI classification, leading to incorrect cell individualization. We set an efficient segmentation scale of 4 after testing different index values (Figs 2c, 3c: original image of mucosa; Fig. 3d: segmented associated image), so that the ROIs were correctly detected and classified (See Fig. 3e: automatic ROI classification; 3f: ROI reclassification by algorithm; 3g: manual correction).

**Figure 4:**
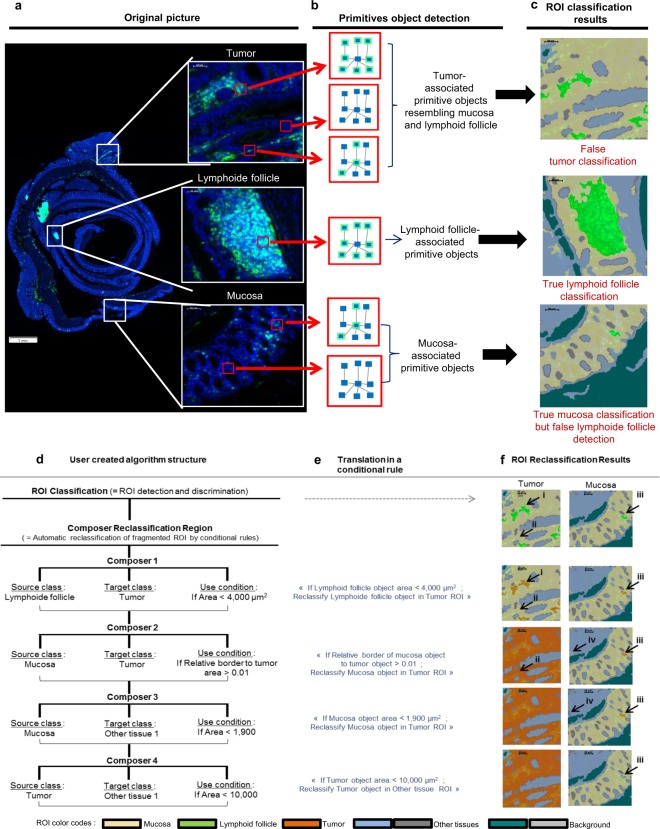
Application of Cognition Network Technology (CNT) on colon ROI classification and user driven improvements. (**a**) An analysis slide is shown with its original picture and specific blow ups corresponding to mucosa, lymphoid tissue and tumor (white boxes), and (**b**) exemplary red boxes pointing out a pixel group as a “primitive-object”. Each of them is automatically associated with a specific ROI, by a stock of ROI-representative “primitive-objects” (**c**, ex: mucosa: yellow area; lymphoid follicle: green area), that were constituted by selecting specific colon area during the algorithm development. With this automatic learning method, we can distinguish lymphoid follicle and mucosa ROI correctly, due to clearly distinct mucosa and lymphoid follicle associated-primitive-objects (**b**,**c**: middle and bottom boxes). On the contrary, tumor-extracted primitive-objects are associated with mucosa and lymphoid follicle-primitive objects (**b;** top boxes), resulting in an incorrect tumor ROI classification (**c**; top box). (**d**–**f**) To solve this issue, we implemented a set of user defined commands to obtain a correct ROI classification. (**d**) We implemented four conditional rules by selecting a ROI couple (the “source class” is the false ROI that needs to be reclassified and the “target class” is the true ROI) and the parameter used to reclassify the ROI. (**e**) Conditional rule selected is translated by the software in a coded rule and (**f**) allows the automatic reclassification of false ROI by software. By using this method we manage to reclassify “false lymphoid follicle” into “tumor”, using a conditional rule based on size object; (**d**–**f**; composer 1, arrow I, II and III) and “false mucosa” into “tumor”, using a conditional rule based on object proximity with tumor (**d**–**f**; composer 2, arrows I, II and III). Same method is used to correct other “false classification” due to confusion between mucosa and other tissue (**d**–**f**; composer 3, arrows II, III and IV) or tumor and other tissue (**d**–**f**; composer 4, arrows II, III and IV).

To conduct this ROI classification, we chose to create three iROI classes and two other ROIs for non-interesting colon regions (“other tissue”) and non-tissue areas (“background”) (See Flowchart in Fig. 2c and the results in Fig. 3e–g). First we implemented a classical computer based learning step called “Training action” in Tissue Studio by recording a precise selection of representative FITC and DAPI labelled primitive objects for each ROI, as exemplified in Fig. 4a. In the first step, the software determined ROI-associated “primitive objects” for each distinct ROI, as represented in Fig. 4a,b. Mucosa, lymphoid follicle and background ROIs were correctly classified at the end of the computer based learning section (Figs 3e and 4c). However, it failed to distinguish the mucosa from other tissue ROI (See Fig. 3e) and the tumor from mucosa or lymphoid follicle (Figs 3e and 4c). In that case, the “primitive-objects” were similar between mucosa and other tissues, or between tumor and lymphoid follicle, and mucosa within a small area, respectively (See tumor-associated “primitive objects”, in Fig. 4b, as an example). To solve this issue, we have added an ROI refining sub-step (named “Reclassification” in Tissue Studio) (See details in Fig. 4d–f). It is based on the selection, organisation and calibration of up to eleven pre-coded conditional rules, from the software library (Summary of the possibilities in Supplementary Table 1). Each of these rules is built in the same fashion (See Fig. 4d,e): it allowed the new classification of an incorrectly classified ROI object into its correct ROI class by selecting a specific characteristic of the object (named “Condition”). More precisely, we implemented and set conditional rules based on the ROI size, tissue complexity and relative borders of each improperly classified ROI (Fig. 4d) which were translated by software (Fig. 4e), allowing a better ROI classification on the whole colon (See result for the whole slide in Fig. 3e,f, white arrows and orange tumor ROI; and see details in Fig. 4f(I) and (II) for example of tumor and Fig. 4f(III) and (IV) for example of other tissue ROI).

To finalize the ROI classification step and to validate the whole process, a visual confirmation was performed as well as a possible ROI refining (Fig. 3g, mucosa: yellow; tumor: orange; lymphoid follicle: green).

### Automated cell detection and classification of CD45^+^ cells

**Figure 5:**
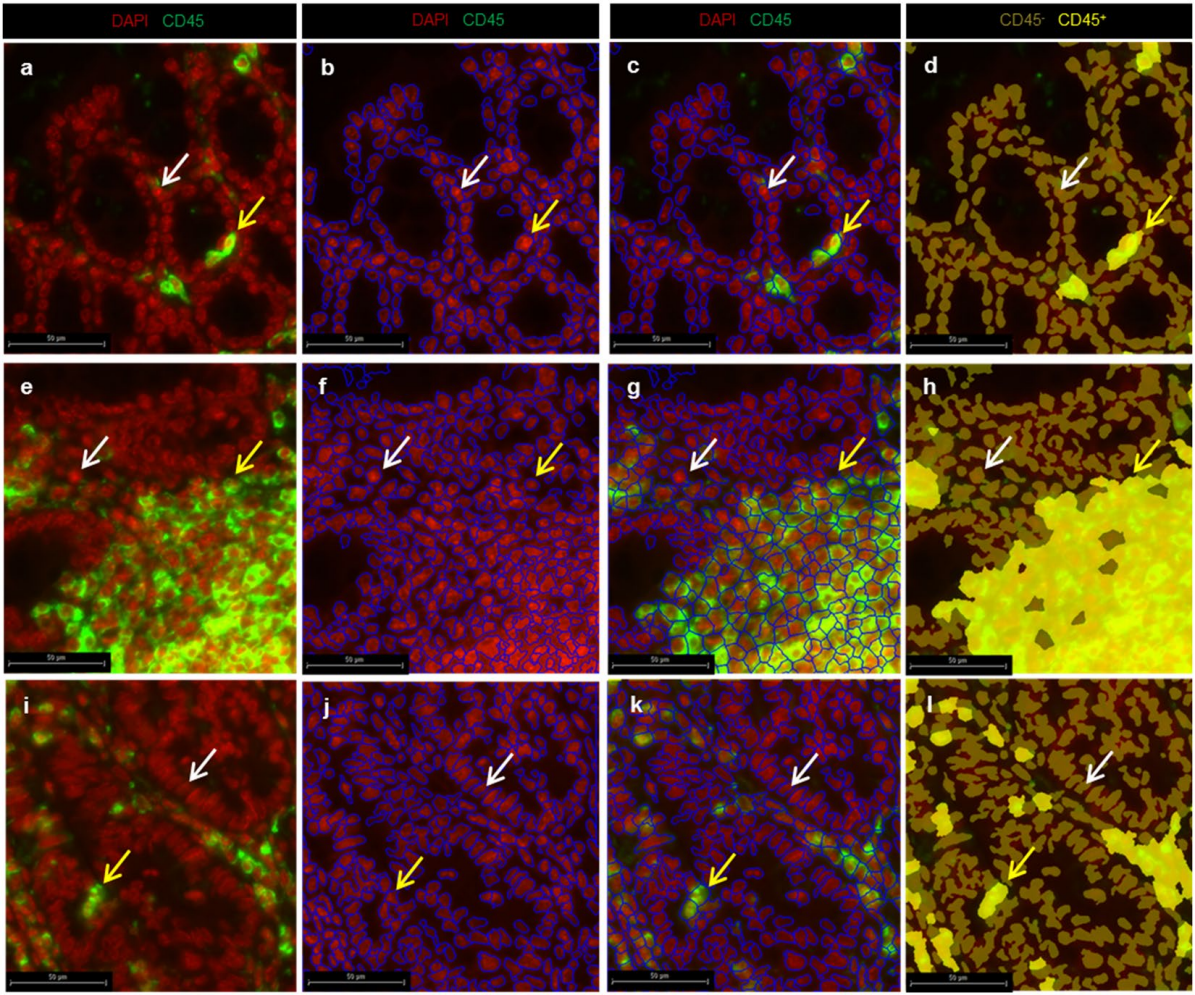
Cellular detection and classification method on mucosa (**a**–**d**), lymphoid follicles (**e**–**h**) and tumor areas (**i**–**l**). (**a**,**e**,**i**) Representative CD45^+^ leucocyte immunostaining (CD45^+^ cells: green; DAPI: red). (**b**,**c**) To detect all cells in the mucosa, we used two sequential steps. (**b**) The first step was nuclear detection as represented by blue delimitation based on DAPI fluorescence and the nuclear mean size as determined by iteration to delimit DAPI (35 µm^2^ ± 5 µm^2^). (**c**) Then, we performed cell detection using a second step with nuclear staining, the CD45 membrane signal and the mean cellular size determined by iteration to delimit FITC staining (38 µm^2^ ± 5 µm^2^). (**d**) To identify leucocytes, we carried out cell classification of positive cells (CD45^+^, light yellow cells and arrows) and negative cells (CD45^−^, white arrow, dark yellow cells), applying optimal fluorescence thresholds for nuclei and membrane markers. We simultaneously performed all of these steps using the same parameters on lymphoid follicles (**e**–**h**) and tumors (**i**–**l**). Automated discrimination of CD45^+^ cells was successful on all iROIs simultaneously with the same optimal parameters.

We performed cell detection in the three iROIs (detailed flowchart in Fig. 2d): mucosa (Fig. 5a–d), lymphoid follicles (Fig. 5e–h) and tumors (Fig. 5i–l). To facilitate cell detection, the algorithm was first set to recognize cell nuclei and cellular membranes and, finally, to simulate cells using the “Inside cytoplasmic stain” mode. This mode was optimal for precise cell delimitation despite the blurry DAPI signal resulting from the high cellular density in the lymphoid follicles as shown in Fig. 5e; and in tumors as shown in Fig. 5i. We successfully programmed the algorithm to recognize different cell types on whole colon tissue, with the same settings for the DAPI fluorescence range and mean nuclear area value. We tested several combinations of these parameters. In this step, the grey value level of DAPI was converted into arbitrary units by Tissue Studio software. We determined the optimal fluorescence range (from 0 to 2 arbitrary units (a.u)) and “mean nuclear area”. This area (35 µm^2^) allowed for individualization and detection of nuclei in each ROI using DAPI signals (Fig. 5b: mucosa, 5f: lymphoid follicle, 5j: tumor). With the same iterative method, we set the CD45 fluorescence threshold at 1,600 grey value levels (g.v.l) and the mean cell area at 38 µm^2^ for optimal cell delimitation. Cell algorithm detection was finally performed and validated for all of the ROIs using these four parameters simultaneously (Fig. 5c: mucosa, 5g: lymphoid follicle, 5k: tumor).

To discriminate CD45^+^ cells (FITC^+^) (Fig. 5, white arrows: negative cells; yellow arrows: positive cells**)**, we implemented a cellular classification step and defined a “positive cell” class based on nuclear and membrane co-staining to avoid incorrect classification due to fluorescence artefacts (e.g., fluorochrome aggregates or red blood cells). Preliminary DAPI and FITC thresholds were set on the basis of the minimal DAPI intensity from 7 random fields of five different colon samples. The maximum FITC intensity was based on 5 control slide random fields without a primary anti-CD45 antibody. We adjusted these parameters after performing simulations to a unique optimal threshold of 1,000 g.v.l for DAPI staining and 1,300 g.v.l for FITC staining (maxima at 65,536 g.v.l). Thus, CD45^+^ cells could be automatically and correctly detected in each iROI for all slides (Fig. 5d: mucosa, 5h: lymphoid follicle, 5l: tumor; yellow cells).

### Validation of the CD45^+^ cell quantification

**Figure 6:**
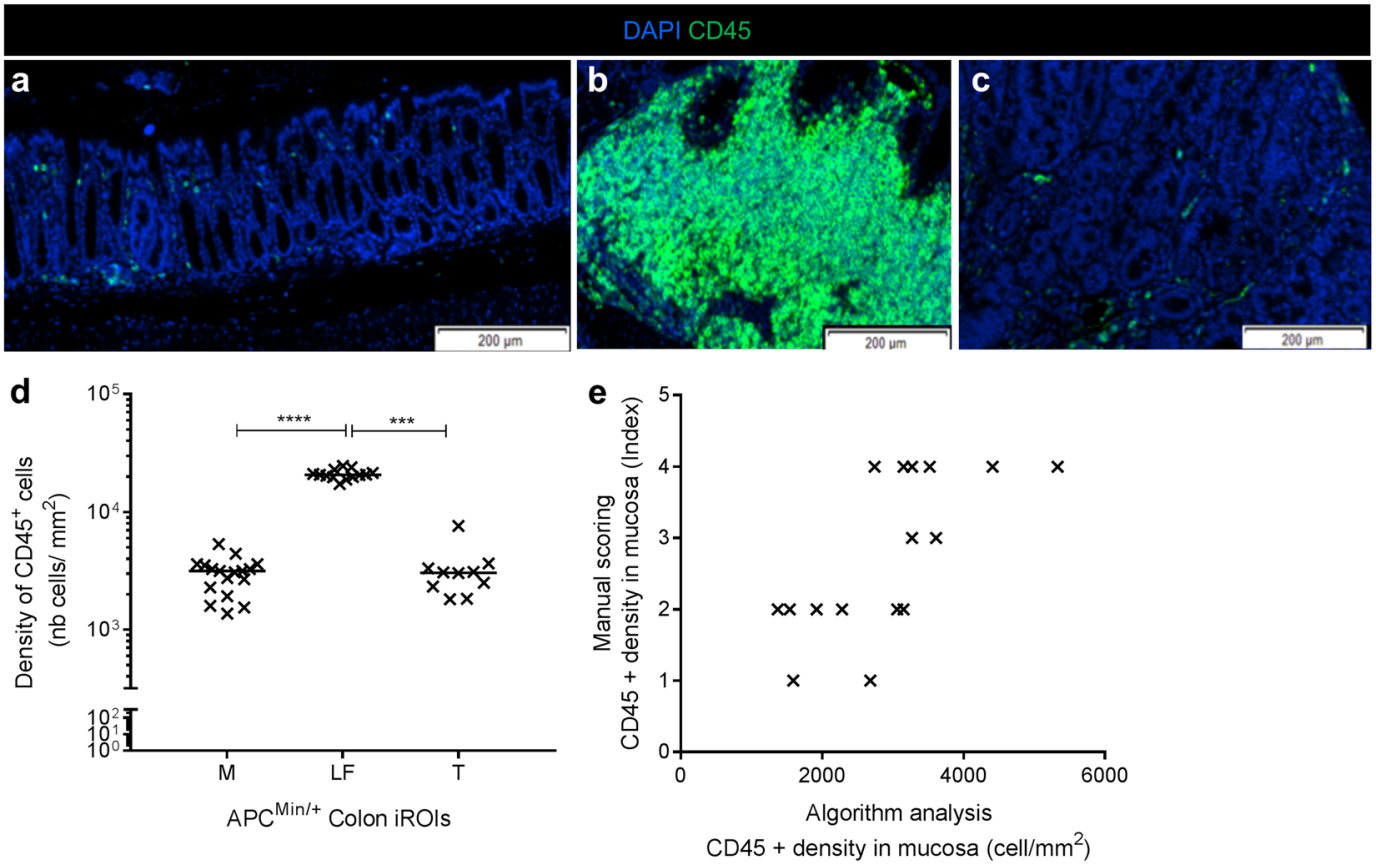
Validation of CD45 leucocyte quantification. (**a**–**c**) CD45 immunostaining for each colon area. As expected, we observed a higher leucocyte density in lymphoid follicle (**b**) than the mucosa (**a**) or tumors (**c**). (**d**) We obtained the same result using the cell quantification algorithm; thus, we validated our method (****p < 0,0001; ***p < 0,005, one-way analysis of variance). (n = 20 immunostained sections from 18 different mice). (**e**) Comparison between algorithm quantification of CD45^+^ cells in the mucosa and manual scoring showed a positive correlation (**p < 0,01; Pearson’s test, r = 0,6869).

Quantification was performed on 20 immunostained whole colon sections (including 2 negative controls) from 18 different APC^Min/+^ mice (Figs 5 and 6), confirming the robustness of our method for simultaneous analysis of a slide batch. Numerical data, such as the iROI area, total cell number and positive cell number per iROI, were exported (See flowchart in Fig. 2e) and cell density was calculated using equation (1).

1 $$\begin{array}{rcl}CD45\,positive\,cell\,densit{y}_{iROIx}(m{m}^{2}) & = & \frac{Total\,CD45\,positive\,cells\,iRO{I}_{x}\,}{Total\,surface\,iRO{I}_{x}\times {10}^{6}}\\  & = & \frac{\#\,Cell\,Markers\,1\,and\,2\,coexpressed\,}{iRO{I}_{x}\,({\rm{all}})\,{\rm{Area}}\times {10}^{6}\,({\mu m}^{2})}\,\,({\rm{Algorithm}}\,{\rm{class}})\end{array}$$

As expected, no positive cells were counted on negative control slides. Moreover, as observed on immunostained APC^Min/+^ mouse colons (Fig. 6a–c), the leucocyte density was far higher in lymphoid follicles (2.10 × 0^4^ cells/mm^2^ ± 0.06 × 10^4^ cells/mm^2^) than in the mucosa (2.97 × 10^3^ cells/mm^2^ ± 0.25 × 10^3^ cells/mm^2^) or tumors (3.22 × 10^3^ cells/mm^2^ ± 0.52 × 10^3^ cells/mm^2^) (See Fig. 6d), showing numeric data consistency. As expected for each whole section, we were able to analyse a significant tissue area per animal, from 0.13 mm^2^ ± 0.03 mm^2^ (for the smallest iROI: lymphoid follicle) to 4.73 mm^2^ ± 0.28 mm^2^ (for the largest iROI: mucosa), which resulted in a significant number of total analysed cells per animal, from 3.73 × 10^3^ ± 1.17 × 10^3^ (for lymphoid follicles) to 1.17 × 10^5^ ± 0.07 × 10^5^ (for the mucosa). Therefore, this method enabled analysis of a significant number of total cells to obtain precise densities of frequent and rare interesting cells in each ROI (Fig. 6b,d).

Thus, we were able to precisely quantify cells in each iROI, a result that was either imprecise by manual scoring or impossible by manual counting due to the high cellular densities in the iROIs. In this way, we were able to compare leucocyte distribution in the three APC^Min/+^ mouse gut immune areas: mucosa, lymphoid follicles and tumors (Fig. 6d, p < 0.0001).

To confirm CD45^+^ leucocyte quantification, we compared the cell density obtained from the digital algorithm measurement (positive cells_iROI_/area_iROI_ (mm^2^)) to the density index assessed by manual scoring in each iROI, as it is presented in Fig. 6e (e.g., mucosa). The range of the scoring index was between 0 (no positive cells) and 5 (elevated positive cell density) and was specific to each iROI. The comparison between manual scoring and the DIA density in the mucosa (Fig. 6e, Pearson r = 0.6869, p < 0.01), tumors and lymphoid follicles (data not shown) highlighted a significant positive correlation that supported the consistency of the numeric data from the algorithm.

### Method application on double staining: CD3^+^ and CD4^+^ T cells

**Figure 7:**
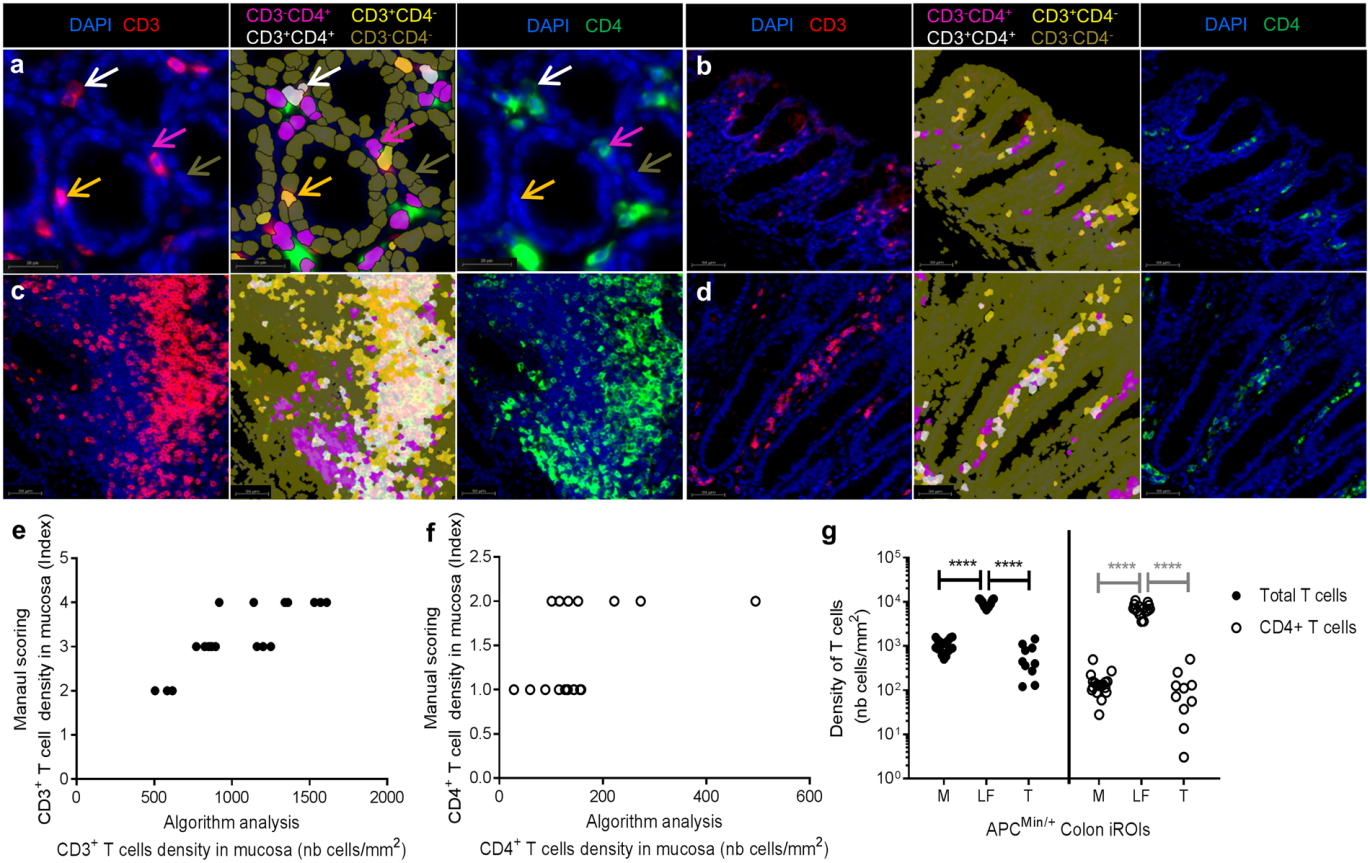
Algorithm application on double immunostaining to quantify CD3^+^/CD4^+^ T cells. (**a**) Representative focus in the mucosa of CD3 (left image; red) and CD4 (right image; green) immunostaining. We based the cell classification on DAPI (blue) and CD3 and CD4 fluorescence to identify four distinct types of cells: CD3^−^CD4^+^ (pink), CD3^+^CD4^−^ (light yellow), CD3^+^CD4^+^ (white) and CD3^−^CD4^−^ (dark yellow) (middle image). (**b**–**d**) We validated our classification method and optimal sets on all iROIs: mucosa (**b**), lymphoid follicles (**c**) and tumors (**d**). (**e**) Comparisons between the algorithm quantification of CD3^+^ cells on the mucosa and manual scoring showed a significant correlation, (****p < 0,0001; Pearson’s test, r = 0,8252). (n = 23 immunostained sections from 18 different mice). (**f**) We obtained a significant correlation for CD3^+^CD4^+^ T cells between the manual and algorithm quantifications (*p < 0,05; Pearson’s test, r = 0,4999) (**g**) We successfully automated the discrimination and quantification of CD3^+^ T cells (fill dot) and CD4^+^CD3^+^ T cells (empty dot) on all ROIs simultaneously using the same parameters. A significant increase of the CD3^+^ T cell density (****p < 0, 0001; one-way analysis of variance) and CD4^+^CD3^+^ T cells (****p < 0, 0001; one-way analysis of variance) was observed in the lymphoid follicle, as expected.

We adapted the same algorithm to analyse double CD3^+^CD4^+^ T cell immunostaining (See flowchart in Supplementary Fig. S2), in which CD3 and CD4 membrane proteins were revealed by Cy3 (Fig. 7a, red cells) and FITC (Fig. 7a, green cells) fluorochromes, respectively. Cell nuclei were counterstained with DAPI (Fig. 7a–d: blue stain).

Because the fluorescence pattern was different from that of CD45, we had to optimise the main parameters of the algorithm (Fig. S2). We adjusted the ROI classification by computer based learning and ROI reclassification rules (See details in Fig. S4, parameter differences between single and double staining are highlighted in blue). Since CD3 is more frequently detected on T cells than CD4, we used the former for cellular delimitation. The optimal fluorescence thresholds (DAPI: from 0 to 2 a.u.; Cy3: from 48,356 to 65,536 g.v.l) and mean areas (nuclei: 35 µm^2^; cell: 36 µm^2^) were applied. Cell classification was modified using DAPI^+^CD3^+^ and DAPI^+^CD3^+^CD4^+^ cell classes for the successful detection of T cells and CD4^+^ T cells, respectively (Fig. 7a). We improved several classification parameters to prevent false CD3^+^ cell detection due to paraffin-embedded tissue edge fluorescence properties (see the Troubleshooting section). As for the CD45 analysis, we used unique fluorescence thresholds (DAPI: 500 g.v.l; Cy3: 2, 500 g.v.l; FITC: 1,500 g.v.l; maxima at 65,536 g.v.l) to detect and classify these cell populations in each iROI (Fig. 7b: mucosa, 7c: lymphoid follicle, 7d: tumor). We exported the same numeric data as in the CD45 analysis and added three export rules to obtain CD4^+^ T cell quantification in each iROI (Supplementary Fig. S2), and cells densities were calculated using equations (2) and (3).

2 $$\begin{array}{rcl}CD3\,positive\,cell\,densit{y}_{iROIx}(m{m}^{2}) & = & \frac{Total\,CD3\,positive\,cells\,iRO{I}_{x}}{Total\,surface\,iRO{I}_{x}\times {10}^{6}}\\  & = & \,\frac{\#\,Cell\,Markers\,1\,and\,2\,and\,3\,coexpressed}{iRO{I}_{x}\,({\rm{all}})\,{\rm{Area}}\times {10}^{6}\,({\mu m}^{2})+iRO{I}_{xbis}\,(all)\,Area\,({\mu m}^{2})\times {10}^{6}}\,({\rm{Algorithm}}\,{\rm{class}})\end{array}$$

3 $$\begin{array}{rcl}CD4\,positive\,cell\,densit{y}_{iROIx}(m{m}^{2}) & = & \frac{Total\,CD4\,positive\,cells\,iRO{I}_{x}}{Total\,surface\,iRO{I}_{x}\times {10}^{6}}\\  & = & \,\frac{\#\,Cell\,Markers\,1\,and\,2\,coexpressed}{iRO{I}_{x}\,({\rm{all}})\,{\rm{Area}}\times {10}^{6}\,({\mu m}^{2})+iRO{I}_{xbis}\,(all)\,Area\,({\mu m}^{2})\times {10}^{6}}\,({\rm{Algorithm}}\,{\rm{class}})\end{array}$$

As described above, we validated each step of our method (Fig. 7a–d) on a batch of whole immunostained colon slides (n = 23, including 5 controls) from different mice (n = 18). Numeric data were compared via manual scoring (Fig. 7e,f), showing a strong correlation for both CD3^+^ T cell (Fig. 7e, Pearson r = 0.8252, p < 0.0001) and CD4^+^ T cell quantification (Fig. 7f, p < 0.05) in the mucosa as well as the other iROIs (data not shown). As expected, a significantly higher density of these cells was observed in the follicle structure (Fig. 7g, Pearson r = 0.4999, p < 0.0001).

### Troubleshooting

We used immunofluorescence staining to detect specific immune cells because it allows for multiplexed analysis^26^. However, the variability of fluorescence detection (the mean intensity, and the variation of fluorescence intensity around the mean and its standard deviation), due to the intrinsic properties of some tissue structures and cell components (elastin, red blood cells), influenced our DIA results (See Table 1). More precisely, we encountered three difficulties during algorithm development. First, the fluorescence signal could be heterogeneous from one slide to another due to a non-optimal acquisition and/or staining protocol (Table 1. 1, 2, 5, 6). Second, the signal could be heterogeneous on the same slide due to intrinsic tissue properties (Table 1. 2–5). Third, fluorescence artefacts associated with staining conditions or paraffin embedding were observed (Table 1. 6–7).

**Table 1:** Troubleshooting guide.

Trouble ID	Troubleshooting	Analysis step	Possible cause	Solutions/Optimization criteria	
				During Digital Image Analysis	During staining and image acquisition
**1**	Partial tissue detection	Global tissue detection	No homogeneous fluorescence signal on whole sections (Intensity, Brightness…)	Choose large thresholds of homogeneity and brightness (0–5 and 0–255)	Use mounting media and a specific IF coverslip (Thickness = 0.17 µm)
					Image acquisition must be processed 1 to 10 days after staining
**2**	Background or other tissue have been incorrectly classified in the mucosa ROI, for a large area of section	ROI classification	Poor separation between the specific signal and background	Use ROI reclassification based on the fluorescence signal intensity, compactness and density of tissue Manual correction step	Optimization of the exposure time and fluorescence minimal and maximal intensity during image acquisition
					All slides must be stained and scanned with the same procedure and same time
			Imprecise automatic learning due to a heterogeneous fluorescence pattern	Make a new automatic learning algorithm using more representative image objects (segmented part of a ROI) from each ROI	
**3**	Background or other tissue have been incorrectly classified in the mucosa ROI for a small area of section	ROI classification	Imprecise automatic learning due to a heterogeneous fluorescence pattern	Make a new automatic learning algorithm using more representative image objects from each ROI	
			Heterogeneous tissue staining or fluorescence pattern for the same ROI	Use ROI reclassification based on the tissue size and relationship to neighbour object	
				Manual correction	
**4**	Lymphoid structure has been incorrectly classified as mucosa	ROI classification	Imprecise automatic learning due to a heterogeneous fluorescence pattern	Make a new automatic learning algorithm using more representative image objects from each ROI CAUTION: DO NOT USE follicle objects with weak cell density for correct follicle area distinction	
			Heterogeneous tissue staining or fluorescence pattern for the same ROI	Use ROI reclassification based on the tissue size and relationship to neighbour object	
				Manual correction	
**5**	Imprecise nuclei detection	Cellular detection	Heterogeneous staining or too blurry fluorescence signal of DAPI	Increase the threshold of DAPI detection	Optimization of staining (Antibody concentration and incubation time)
					Optimization of automated focus during scan, possibility to apply Extending Focus Imaging)
					Optimization of fluorescence acquisition (exposure time, intensity thresholds)
			Heterogeneous staining or too blurry fluorescence signal of membrane marker	Increase the threshold of membrane marker detection if the fluorescence membrane intensity is too high	
				Decrease the threshold of membrane marker detection if the fluorescence membrane intensity is too low	
				Use the detection mode “growth from nuclei”	
**6**	False-positive cells detected	Cellular classification	Imprecise cell detection (cf Troubleshooting 5)	Cf Troubleshooting 5	Cf Troubleshooting 5
			Fluorochrome deposits	Increase the threshold of DAPI for cell classification	Optimization of staining (decrease concentration of detection, decrease time of denaturation by heat, increase wash number and time)
				Increase the minimal size of cells during membrane detection step	
				Exclude aggregates of fluorochromes from the cellular analysis using nuclear filter module	
			Tissue autofluorescence on the margin (mucosa and tumor) specific to paraffin-embedded tissue	Exclude area of cellular analysis with the use of new area classification (Mucosa bis, Tumor bis)	Increase the difference between background and specific staining during image acquisition (time exposure, fluorescence intensity thresholds)
**7**	Aberrant cellular detection links with specific membrane or nuclear structures (e.g., polynuclear cells)	Cellular detection	Aberrant distinction between the nuclear and membrane signals due to incorrect membrane detection by the algorithm	Decrease the resolution and magnification parameter analysis and only use membrane staining to detect cells	

All of the steps are based on the fluorescence intensity and fluorescence intensity thresholds, so they were sensitive to any change. This table provides solutions to overcome these problems and quickly optimise the analysis.

To address these phenomena, the staining protocol and image acquisition were optimised (Table 1, sixth column). Importantly, the same conditions can only be applied to a batch of slides when the fluorescence patterns are homogenous on all slides. To this end, it is essential to use the same staining and acquisition parameters (Table 1. 1, 2, 5, 6: sixth column). To obtain optimal signals (homogeneity, minimal and maximal intensity) for DIA, the preferred time to scan a slide batch is up to 10 days after staining. The other optimal parameters are precisely described in the Online Methods.

In cases in which staining and acquisition protocol optimization were insufficient (Table 1. 3, 4, 5, 7), improvements of the DIA algorithm played a key role in preventing analysis errors (see the troubleshooting guide Table 1, fifth column). In particular, fluorescence heterogeneity on the same slide (e.g., due to biological heterogeneous marker distribution or tissue states) caused improper ROI classification. We overcame the inability of Tissue Studio to determine a general fluorescence pattern for each area by adding reclassification rules based on fluorescence-independent parameters, such as the ROI size or tissue complexity (Table 1. 2–4). Fluorescence artefacts inducing false positive cell detection (1.9% of slides for a single staining, or 30.4% of slides for a double staining) could also be solved by minor algorithm modifications. This correction step was more easily achieved if the affected ROI did not contain target cells (Table 1. 6).

Provided attention is paid to these critical points, the pipeline we describe here allows immune colon profiling on numerous slides without failure. It is important to mention that the dependence of the algorithm on membrane detection means that it must be optimised for each new surface marker. However, this can be accomplished quickly by adjusting the numeric parameters according to the recommendations of our troubleshooting guide (Table 1).

## Discussion

Here, we describe a new quantitative semi-automated method to characterize the immune microenvironment on whole mouse colon slides. Based on CNT, the proposed method allows tissue to cell scale analysis; thus, we are able to simultaneously investigate various ROIs and immune cell populations associated with colonic tissue. A recent immune cell quantification study by Blom and colleagues used another software on a slide of prostate mice tumor^26^ but did not distinguish different structures as with our method using ROI classification. Here, we show how to use our method to quantify leucocytes and T cells in three immune colon regions in APC^Min/+^ mouse model (mucosa, lymphoid follicle and tumor), and it is possible to implement up to 8 ROIs. This new analysis enables precise quantification of cells and comparison of cell distributions between different areas in whole tissue.

Compared to classical IHC scoring, this method using automatic learning avoids human bias and quantifies cells quickly without laborious manual work. Moreover, as the whole tissue and all the cells are analysed on the slide (from 0.13 to 4.73 mm^2^ of tissue and from 10^3^ to 10^5^ cells), we provide accurate results compared to classical IHC assessment in a few random fields^13^. Therefore, this new method can consider biological variations and rare cell populations.

Our method can act as a complementary tool to flow cytometry (FC) because it allows analysis of significant cell number without requiring a complex and laborious cell extraction process. Cross-correlation and cellular relationships between immune cell density and other staining methods (*e.g*., FISH, HES, cellular damage staining) performed on consecutive sections are possible, adding new possibilities compared to classical FC studies.

Furthermore, thanks to the development of our algorithm, its step by step description, and its troubleshooting guide (Table 1), it is easier to analyse data than to perform mass spectrometry imaging. If necessary, our algorithm can be easily applied to the Definiens software interface using the Tissue Studio User guide^29^. Additionally, obtaining numerical data is faster than with mass spectrometry or classical IHC. Thus, we developed a fast and simple way to robustly quantify and localize immune cells on numerous tissue samples without the requirement of complex bioinformatics expertise.

Moreover, our method allows the cell density to be determined from microscopic structures, such as lymphoid follicles and small neoplastic lesions, which are not accessible with FC. Because of the Definiens CNT used in our algorithm, we can implement specific settings to detect these structures within the colon and count the associated cells. This gives significant information as the high density of immune cells in lymphoid structures shows the link between gut inflammation and lymphoid follicles, which has already been described in the mouse^30^; furthermore, the importance of the tumor microenvironment has been shown in colorectal cancer patients^6,7,18^.

Because our algorithm is based on immunofluorescence detection, its settings are sensitive to fluorescence variations (e.g., pattern and intensity). Whereas the method pipeline is validated on different slide sections and stainings, an optimal and reproducible immunostaining protocol is required to analyse a batch of slides. Furthermore, algorithm optimization is needed for each new immunostaining or to develop new membrane markers. A simple software interface^28,29^ and troubleshooting guide make this process easier and faster for everyone.

Application of this new, quantitative, easy method could be extended to multiplex studies, such as the work by Blom^26^, as well as to whole sections with various complex structures, such as gut samples, because these settings are optimised for this type of tissue. Application of this method enables precise immune cell characterization in various gut regions of interest (from the epithelium to muscular layers) via cell-cell interaction studies and fine localization of cellular events at different time points along the gut. This method could be useful to better understand both the mechanism of immune dysfunction associated with gut disorders^1–8^ and the interaction between pathogenic bacteria (FISH determination) and the immune system^9,10,12,22^.

In summary, here, we describe a powerful method to characterize immune cells in the gut of mice that is user-friendly for the scientific community. We expect that this method will help to decipher the complexity of interactions between the gut microenvironment and gut disorders or their treatments. Moreover, this method could apply to the detection and quantification of cells in any particular microscopic structure that is not reachable by classical means. For example, subtle neuronal immune cross talk in the gut, such as macrophages interacting with mesenteric neurons^31^ could be studied using the developed algorithm and method in a quantitative fashion. It will allow scientist to follow the precise and quantitative development of specific microenvironment within a disease context in whole organs for the purposes of preclinical studies.

## Methods

### Animals

Studies were performed using 13- to 14-week-old C57BL/6J-ApcMin/þ (Min mice; The Jackson Laboratory, Bar Harbor, ME, USA) in accordance with the French and European Economic Community guidelines (86-60, EEC) for the care of laboratory animals. Studies were approved by the French Regional Ethical Animal Use Committee (No. CE-2912, Apafis#5401). All mice were housed in specific pathogen-free conditions at the animal care facility of Université Clermont Auvergne (Clermont-Ferrand, France).

### Tissue harvesting and processing

The colons were removed from the caecum to the rectum, flushed with PBS, and splayed longitudinally. The colons were swiss-rolled from the distal to proximal end and fixed for 24 h in 10% formalin (Sigma, Tokyo, Japan) at room temperature (RT). Colon blocks were embedded in paraffin (Tissue-Tek Paraffin Wax #4511, Sakura, Leiden, Holland) according to the standard procedure. Paraffin-embedded sections were cut into 5 µm sections on superfrost slides (Thermo Scientific^TM^, Waltham, MA, USA), dried overnight at +37 °C and stored at RT. For long-term storage, slides were stored at +4 °C.

### Immunofluorescence staining (IF) and mounting slides

All IF staining was performed using an automated stainer, Discovery XT processors (Ventana Medical Systems, Oro Valley, Arizona, USA), and the tyramide signal amplification (TSA)-conjugated fluorochrome method was used to detect each target (CD45, CD3 and CD4) on whole colon slides. Paraffin was removed from slides, and slides were then conditioned with two incubation cycles in EZPrep buffers (Ventana Medical Systems, Oro Valley, Arizona, USA), with a first cycle at 58 °C for 12 min and a second cycle at 60 °C for 4 min. Antigens were retrieved with CC1 program (Ventana Medical Systems, Oro Valley, Arizona, USA) according to the standard procedure (Tris-EDTA buffer pH 7.8 at 95 °C for 44 min). Then, endogenous horseradish peroxidase was blocked with DISC inhibitor reagent (Ventana Medical Systems, Oro Valley, Arizona, USA) for 8 min at RT.

A series of staining steps were performed interspersed with 2 to 3 washing steps using the proprietary Reagent Buffer (Ventana Medical Systems, Oro Valley, Arizona, USA). For CD45 single staining, slides were incubated with primary rabbit anti-CD45 antibody (10558, 2.5 µg/mL; Abcam, Cambridge, UK) at RT for 2 h. For IF, two rounds of 16 min incubations were performed, first with Umap anti-rabbit HRP (Ventana Medical Systems, Oro Valley, Arizona, USA) and second with FITC tyramides and H_2_O_2_ (Discovery FITC kit, Ventana Medical Systems, Oro Valley, Arizona, USA) according to the manufacturer’s instructions.

For CD4/CD3 double staining, staining was performed in three sequential steps. First, slides were incubated with a primary rat anti-CD4 antibody (4SM95 clone, 2.5 µg/mL, eBioscience, Paris, France) at RT for 1 h. For IF, slides were incubated with a goat anti-Rat IgG secondary antibody (112-065-003, 5.5 µg/mL; Jackson,Baltimore, PA, USA) for 32 min at RT then incubated with Umap anti-goat HRP (Ventana Medical Systems, Oro Valley, Arizona, USA) according to the manufacturer’s instructions for 16 min at RT. Finally, they were reacted with FITC tyramides and H_2_O_2_ (Discovery FITC kit, Ventana Medical Systems, Oro Valley, Arizona, USA) for 16 min at RT. A second step comprising denaturation and neutralization was needed for CD3 staining. More precisely, slides were incubated at 40 °C for 20 min with DISC inhibitor HRP reagent (Ventana Medical Systems, Oro Valley, Arizona, USA). Third, they were incubated with a primary rabbit anti-CD3 antibody (SP7 clone, Thermo Fisher, Waltham, MA, USA, 5 µg/mL) at RT for 2 h. For IF, slides were incubated with Umap anti-rabbit HRP (Ventana Medical Systems, Oro Valley, Arizona, USA) according to the manufacturer’s instructions for 16 min at RT. Finally, a reaction between Rhodamine tyramides and H_2_O_2_ (Discovery Rhodamine kit, Ventana Medical Systems, Oro Valley, Arizona, USA) was performed for 16 min at RT.

The cut sections were denatured at 37 °C for 4 min, and cell nuclei were counterstained with DAPI (Ventana Medical Systems, Waltham, MA, USA,). Slides were manually mounted with ProLong™ Gold Antifade Mountant (Thermo Fisher, Waltham, MA, USA) and High Precision coverslips (No 1.5 H, Marienfeld, Thuringia, Germany), which are optimal for optical microscopy acquisition.

Negative control slides were also included that lacked any appropriate antibody or, in the case of double staining, omitted one or the other primary antibody.

### Image acquisition

All slides were acquired automatically at 0,161 µm/pixel resolution using a VS-120-ASW-L100 whole-slide scanner (Olympus, Rungis, France) equipped with a Digital monochrome Olympus XM10 camera 40x objective (NA 0.95). First, a global overview of the whole colon section was performed automatically using an Olympus camera set on brightfield (VC50 camera 4x objective and the mercury lamp U-HGLPS, Lumencor) to define the scan area. Second, a fluorescence image was acquired with automatic focusing (default factors setting) and used DAPI (ex: 350 nm/em: 450 nm), FITC (ex: 495 nm/em: 521 nm) and CY3 (for Rhodamine fluorophore, ex: 550 nm/em: 570 nm) filter sets compatible with light sources (X-Cite exacte Microscope Illumination System) and AT350/50x filter wheels. For CD45 fluorescence imaging, we used 50 ms optimal exposure times for the DAPI channel and 25 ms for the FITC channel. For CD3/CD4 fluorescence staining, we used 30 ms optimal exposure times for the DAPI channel, 50 ms for the FITC channel, and 5 ms for the CY3 channel. Fluorescence images were stored as 16-bit greyscale fields and global overview images as 24-bit RGB colours. All images were acquired with Olyvia VS-ASW FL 2.7 software (Olympus, Rungis, France; build number 11043) and stored under an *ets* format linked to an Olympus proprietary *vsi* file.

### Manual scoring

Manual scoring was performed on Olyvia VS-ASW FL 2.7 software (Olympus, Rungis, France; build number 11043) on a Z840 HP PC with a 4 cores Intel Xeon CPU E5-2637 V4 (3.50 GHz), 128 GB of RAM, and Windows 7 Entreprise (64 bit). Analyses were performed on 20 different whole colon sections from 18 different mice for CD45 staining (18 slides with IF staining and 2 negative controls without anti-CD45 primary antibody). For T cell analysis, we used the same 18 mice, but we performed analyses on 23 different sections (18 IF stains and 5 controls: 3 without anti-CD3 and CD4 primary antibodies, 1 without anti-CD3 primary antibody, and 1 without anti-CD4 primary antibody). To score leucocytes (CD45^+^ cells) and CD3 and CD4 T cell densities, the iROI (mucosa, lymphoid follicle and tumor) of each mouse was observed with a 20x objective and scored separately. For mucosa and tumor, 10 and 7 random fields were observed and scored, respectively, and then the mean was calculated. For lymphoid follicles, the whole lymphoid area was observed. The manual score was determined between 0 (absence of positive immune cells) and 5 (high presence of positive immune cells). The slides were anonymously analysed; to simplify scoring, each specific iROI (mucosa, lymphoid follicle, and tumor) was compared between the different mice, but the iROIs of the same mouse were not compared with each other.

### Digital image analysis and code availability

Digital image analysis was performed on the same hardware as the manual analysis, using the IF module of Definiens Tissue Studio software version 4.3.1. All images were downloaded in the original format (*ets*, 0.161 µm/pixel resolution; 16-bit depth, 40x objective). Each slide was automatically analysed using 40x viewing resolution for segmentation and cellular analysis steps, and 10x viewing resolution was used for ROI detection and classification steps. Across the entire analysis between ROI correction and cellular analyses steps, the same resolution parameters were selected. Each step was confirmed visually. To obtain the best distinction between the algorithm simulation and the original image, we used a false colour representation (DAPI in red, membrane marker in green). First, algorithm settings (described previously) were established on five representative slides. They then were tested and optimised on 10 randomly selected slides. Finally, they were confirmed on each batch of slides (20–23 slides). Validated algorithm codes were saved as *dax* files that are available at Scientific Report online.

### Numeric data analysis and statistical methods

The area of each iROI was directly exported by Tissue Studio software using Microsoft Excel tables (Microsoft office professional Plus 2010; Redmond, Washington, USA). CD45 and T cell densities on each iROI were calculated with Excel:

$$\begin{array}{rcl}CD45\,positive\,cell\,densit{y}_{iROIx}(m{m}^{2}) & = & \frac{Total\,CD45\,positive\,cells\,iRO{I}_{x}}{Total\,surface\,iRO{I}_{x}\times {10}^{6}}\\  & = & \frac{\#\,Cell\,Markers\,1\,and\,2\,coexpressed\,}{iRO{I}_{x}\,({\rm{all}})\,{\rm{Area}}\times {10}^{6}\,({\mu m}^{2})}\end{array}$$

$$\begin{array}{rcl}CD3\,positive\,cell\,densit{y}_{iROIx}(m{m}^{2}) & = & \frac{Total\,CD3\,positive\,cells\,iRO{I}_{x}}{Total\,surface\,iRO{I}_{x}\times {10}^{6}}\\  & = & \frac{\#\,Cell\,Markers\,1\,and\,2\,and\,3\,coexpressed\,}{iRO{I}_{x}\,({\rm{all}})\,{\rm{Area}}\times {10}^{6}\,({\mu m}^{2})+iRO{I}_{xbis}\,(all)\,Area\,({\mu m}^{2})\times {10}^{6}}\end{array}$$

$$\begin{array}{c}\begin{array}{rcl}CD4\,positive\,cell\,densit{y}_{iROIx}(m{m}^{2}) & = & \frac{Total\,CD4\,positive\,cells\,iRO{I}_{x}}{Total\,surface\,iRO{I}_{x}\times {10}^{6}}\\  & = & \frac{\#\,Cell\,Markers\,1\,and\,2\,coexpressed\,}{iRO{I}_{x}\,({\rm{all}})\,{\rm{Area}}\times {10}^{6}\,({\mu m}^{2})+iRO{I}_{xbis}\,(all)\,Area\,({\mu m}^{2})\times {10}^{6}}\end{array}\end{array}$$

Statistical analyses were performed using Prism 6.05 (GraphPad Prism, La jolla, CA, USA). Correlations between manual score and numeric data analyses were calculated using Pearson’s correlation coefficient (r). One-way analysis of variance test was used to compare cell density between iROIs of the same mouse. p-values <0.05 were considered statistically significant.

### Accession code

Algorithm codes for digital image analysis of whole colon slide, and more precisely for ROI classification and leucocytes and T cells quantification are available on Scientific Report website, in *dax* format. This format must be opened in Tissue Studio Software. Structures and all any details of algorithm code are available in.pdf format in article figures (Figs 1a, 2 and 4d) and in Supplementary information (Supplementary Figs 2 and 3).

## Electronic supplementary material

Supplementary Information

## References

[1] Yan Y (2009). Temporal and Spatial Analysis of Clinical and Molecular Parameters in Dextran Sodium Sulfate Induced Colitis. PLoS ONE.

[2] Hall LJ (2011). Induction and Activation of Adaptive Immune Populations During Acute and Chronic Phases of a Murine Model of Experimental Colitis. Dig. Dis. Sci..

[3] Lissner, D. *et al*. Monocyte and M1 Macrophage-induced Barrier Defect Contributes to Chronic Intestinal Inflammation in IBD: *Inflamm*. *Bowel Dis*. **1**, 10.1097/MIB.0000000000000384 (2015). 10.1097/MIB.0000000000000384 PMC4450953 25901973

[4] Kim D, Zeng MY, Núñez G (2017). The interplay between host immune cells and gut microbiota in chronic inflammatory diseases. Exp. Mol. Med..

[5] Tosolini M (2011). Clinical Impact of Different Classes of Infiltrating T Cytotoxic and Helper Cells (Th1, Th2, Treg, Th17) in Patients with Colorectal Cancer. Cancer Res..

[6] Galon J (2012). Cancer classification using the Immunoscore: a worldwide task force. J. Transl. Med..

[7] Mlecnik B (2016). Integrative Analyses of Colorectal Cancer Show Immunoscore Is a Stronger Predictor of Patient Survival Than Microsatellite Instability. Immunity.

[8] Jakubowska Katarzyna, Kisielewski Wojciech, Kańczuga-Koda Luiza, Koda Mariusz, Famulski Waldemar (2017). Diagnostic value of inflammatory cell infiltrates, tumor stroma percentage and disease-free survival in patients with colorectal cancer. Oncology Letters.

[9] Wu S (2009). A human colonic commensal promotes colon tumorigenesis via activation of T helper type 17 T cell responses. Nat. Med..

[10] Kostic AD (2013). Fusobacterium nucleatum Potentiates Intestinal Tumorigenesis and Modulates the Tumor-Immune Microenvironment. Cell Host Microbe.

[11] Yu Y-RA (2016). A Protocol for the Comprehensive Flow Cytometric Analysis of Immune Cells in Normal and Inflamed Murine Non-Lymphoid Tissues. PLOS ONE.

[12] Thiele Orberg E (2017). The myeloid immune signature of enterotoxigenic Bacteroides fragilis-induced murine colon tumorigenesis. Mucosal Immunol..

[13] Mulrane L, Rexhepaj E, Penney S, Callanan JJ, Gallagher WM (2008). Automated image analysis in histopathology: a valuable tool in medical diagnostics. Expert Rev. Mol. Diagn..

[14] David BA (2016). Combination of Mass Cytometry and Imaging Analysis Reveals Origin, Location, and Functional Repopulation of Liver Myeloid Cells in Mice. Gastroenterology.

[15] Newell EW, Cheng Y (2016). Mass cytometry: blessed with the curse of dimensionality. Nat. Immunol..

[16] Chang Q (2017). Imaging Mass Cytometry. Cytometry A.

[17] Eliceiri KW (2012). Biological imaging software tools. Nat. Methods.

[18] Galon, J. *et al*. Immunoscore and Immunoprofiling in cancer: an update from the melanoma and immunotherapy bridge 2015. *J. Transl. Med*. **14**, 10.1186/s12967-016-1029-z (2016). 10.1186/s12967-016-1029-z PMC5029056 27650038

[19] Kozlowski C (2013). An entirely automated method to score DSS-induced colitis in mice by digital image analysis of pathology slides. Dis. Model. Mech..

[20] Moser AR, Pitot HC, Dove WF (1990). A dominant mutation that predisposes to multiple intestinal neoplasia in the mouse. Science.

[21] Levy, D. B. *et al*. Inactivation of both APC alleles in human and mouse tumors. *Cancer Res*. **54**, 5953–5958, http://cancerres.aacrjournals.org/content/54/22/5953 (1994). 7954428

[22] Mima K (2015). *Fusobacterium nucleatum* and T Cells in Colorectal Carcinoma. JAMA Oncol..

[23] Jochems C, Schlom J (2011). Tumor-infiltrating immune cells and prognosis: the potential link between conventional cancer therapy and immunity. Exp. Biol. Med..

[24] Stack EC, Wang C, Roman KA, Hoyt CC (2014). Multiplexed immunohistochemistry, imaging, and quantitation: A review, with an assessment of Tyramide signal amplification, multispectral imaging and multiplex analysis. Methods.

[25] Feng Z (2016). Multispectral Imaging of T and B Cells in Murine Spleen and Tumor. J. Immunol..

[26] Blom, S. *et al*. Systems pathology by multiplexed immunohistochemistry and whole-slide digital image analysis. *Sci. Rep*. **7**, 10.1038/s41598-017-15798-4 (2017). 10.1038/s41598-017-15798-4 PMC5686230 29138507

[27] Parra, E. R. *et al*. Validation of multiplex immunofluorescence panels using multispectral microscopy for immune-profiling of formalin-fixed and paraffin-embedded human tumor tissues. *Sci. Rep*. **7**, 10.1038/s41598-017-13942-8 (2017). 10.1038/s41598-017-13942-8 PMC5645415 29042640

[28] Baatz M, Zimmermann J, Blackmore CG (2009). Automated Analysis and Detailed Quantification of Biomedical Images Using Definiens Cognition NetworkTechnology. Comb. Chem. High Throughput Screen..

[29] Castellanos JA, Montiel JMM, Neira J, Tardós JD (1999). The SPmap: A probabilistic framework for simultaneous localization and map building. IEEE Trans. Robot. Autom..

[30] Bergomas F (2011). Tertiary Intratumor Lymphoid Tissue in Colo-Rectal Cancer. Cancers.

[31] Veiga-Fernandes H, Artis D (2018). Neuronal-immune system cross-talk in homeostasis. Science.

