# Supplementary material for: Deciphering the immune microenvironment of a tissue by digital imaging and cognition network
Source: Sci Rep. 2018 Nov 12;8:16692. doi: 10.1038/s41598-018-34731-x (PMC6232093; doi:10.1038/s41598-018-34731-x)
Supplement: Supplementary file 1 — Supplementary Information [file 41598_2018_34731_MOESM1_ESM.docx]

**Deciphering the immune microenvironment of a tissue by digital imaging and cognition network.**

A. Lopès^1,2,3^, Al H. Casse^4, +*^, E. Billard^1,2, +^, E. Boulcourt-Sambou^4^, G. Roche^1,2^, C. Larois^4^, N. Barnich^1,2^, S. Naimi^4^, , M. Bonnet^1,2,+^, B. Dumas^3+*^.

^1^ Clermont Université, UMR 1071 Inserm/Université Clermont-Auvergne, 63000 Clermont-Ferrand, France

^2^ INRA, USC-2018, 63000 Clermont-Ferrand, France

^3^ Research Biologics, Sanofi R&D, 94400 Vitry-Sur-Seine, France

^4^ Histopathology and Bio-Imaging Group, Sanofi R&D, 94400 Vitry-Sur-Seine, France

^+^Contributed equally

*Corresponding author

*Bruno.Dumas@sanofi.com // Alhassan.Casse2@sanofi.com

**Supplementary informations**


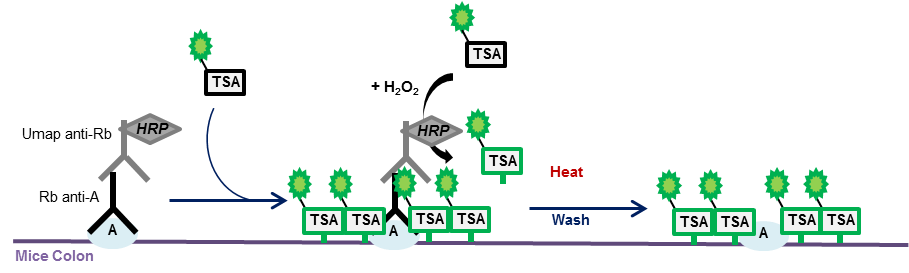
**Supplementary Figures**

**a**


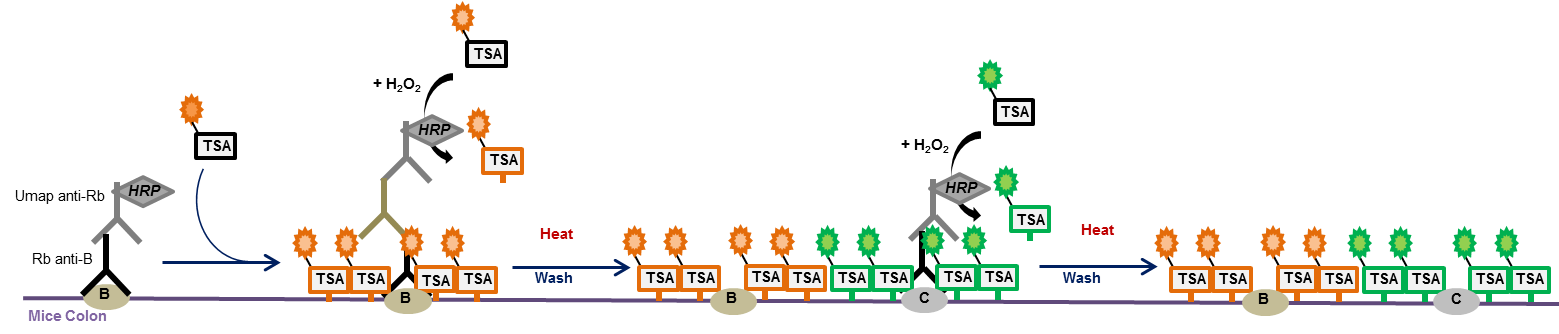


**b**

**Supplementary figure S1.** Optimal immunostaining method for digital image analysis. The Tyramide Signal Amplification method amplifies specific fluorescence signals on IHC colons of APC^Min/+^ mouse slides and thus allows automated digital analysis. This method is based on the reaction between horseradish peroxidase (HRP) and tyramide (TSA) and is used to reveal the detection of immune membrane markers (A,B,C) during simple (a) and double staining (b). Briefly, an unconjugated primary antibody recognizes a protein of interest (A,B,C) and is then recognized by a secondary antibody coupled with Horseradish Peroxidase (HRP). When tyramide coupled with a fluorochrome is added, it reacts with HRP and becomes activated. The tyramide-fluorochrome complex forms a covalent link with the target protein. Finally, the large number of these complexes amplifies the signal. This technique permits more than simple staining because of a denaturation step by heating (b).

**Cell Simulation :** Simulate inside cytoplasmic stain mode

**Composer Reclassification Region** ( = Automatic reclassification of fragmented ROI by conditional rules see Supplementary Figure 6)

**Cell Classification**

Feature selected : Coexpression Marker 1and 2 and 3 (« Class 4 »)

**Tissue Detection** ( = Global tissue detection)

**a**

**General settings** ( = Picture acquisition settings)

Magnification

40x

Pixel Size

0.161 µm/pxl

Bit Depth

16 bits

Fluorescence Channel

- Layer 1 : DAPI = « Nuclei »
- Layer 2 : FITC = « CD4 »
- Layer 3 : Cy3 = « CD3 »

**b**

Use layers

(= Fluorochrome channel selected)

Nuclei, CD4, CD3

Tissue Min Size

10 µm2

Brightness threshold

0 u.a

Homogeneity threshold

0 u.a

**ROI Classification** (= ROI detection and discrimination)

**c**

**Composer initialization** ( = Magnification selected for ROI analysis) : Magnification 10 x

Segmentation scale index

(= picture segmentation level)

Index 3

Sample detection mode

(= ROI discrimination method)

Classify object

Sample selection (= ROI names)

ROI 1 : «Tumor»

ROI 2 : «Lymphoid follicle»

ROI 3 : «Other tissues»

ROI 4 : «Mucosa»

ROI 5 : «Other tissues 2»

ROI 6 : «Background»

ROI 7 : « Mucosa Bis »

ROI 8 : « Tumor Bis »

Use layers

Nuclei

**Composer ROI Correction** ( = Quick manual validation of ROI classification by user)

Turn « Interrupt on server » to « store ROI classification »

**Cellular analysis** ( = Cellular detection and classification)

**d**

Use Nucleus layer

Nuclei

Typical nuclei size

35 µm^2^

**Initialize Cellular Analysis** ( = Settings for cellular analysis)

Magnification 10 x ; ROI selection for cellular analysis : Tumor, Lymphoide follicule , Mucosa

**Nuclei Detection**

Nuclei Stain range

0-2 u.a

User layers

CD3

Typical cellular size

36 µm^2^

Stain threshold

0 g.v.l

Marker 1: Nuclei

Threshold : 500 g.v.l

Class 1 : Nuclei

Marker 2: CD3

Threshold : 2,500 g.v.l

Class 2 : Cell

**e**

**Export**

Custom export ROI analysis

Statistic per slide

Statistic per ROI

Custom export cell analysis

Exported class = Tumor ; Mucosa ; Lymphoide follicle

Features = Area ; Fluorescence intensity mean

Exported class = Nuclei ; Cell ; Cells marker 1 and 2 coexpress ;

Cells marker 1 and 2 and 3 coexpress ;

Features = Size ; Fluorescence intensity mean ; Number ; Sum

Membrane mask

CD3

Membrane Stain maxima limit

48,356 g.v.l

Marker 3 : CD4

Threshold : 1,500 g.v.l

Class 3 : Cell

**Training action**

( = Automated learning based on user selected of ROI-associated colon region)

**Supplementary figure S2.** Optimal digital image analysis algorithm flowchart for whole colon slide, for CD3/CD4 double staining. Total T cells and CD4^+^ T cells were quantified in the same way as leucocytes CD45^+^ cells. Exactly the same orderly rule successions are implemented, the only changes (appear in blue characters in flowchart) are the addition of a new fluorescence channel corresponding to Cy3 fluorochrome (used to reveal the CD3 membrane protein), and the settings of specific parameters based on fluorescence. (**a**) More precisely, the new fluorescence channel is added in the first step, “General settings”. (**b**) We optimize “Tissue detection” by adding the Cy3 channel to detect whole tissue and setting the brightness fluorescence threshold. (**c**) For the “ROI Classification”, minor changes are made. The index of segmentation is decreased to allow a better “primitives-object” formation (determined by iterative method). Besides we add two ROI classes: “Mucosa bis” and “Tumor bis” to class exceptional Mucosa and Tumor ROI without cells of interest but with low auto-fluorescence (in Cy3 fluorescence channel), when it is necessary. It allows measuring the tissue size without counting cells because these ROIs are not selected in final cellular analysis (**d**. “Initialize Cellular Analysis” step). (**d**) Concerning the other “Cellular analysis” steps, we allow the detection and classification of total CD3^+^ T cells and CD4^+^CD3^+^ T cells. To this end we designate the CD3 marker (Cy3 channel) as the principal membrane component by adapting the Nuclei detection and Cell simulation (blue characters). Furthermore we implement a third cell class (blue characters in “Cell Classification” step). (e) Rules of exportation are the same as for CD45 analysis; we simply add an export class to obtain the number of CD3^+^ CD4^+^ T cells (blue characters). Composer = ruleset.


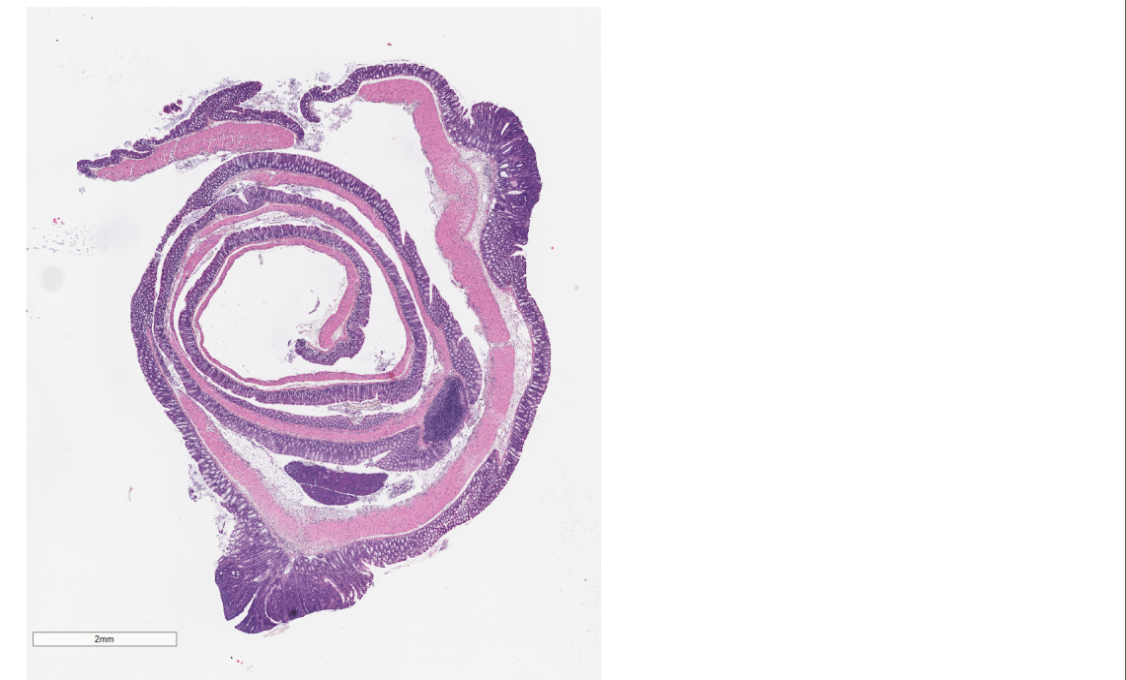

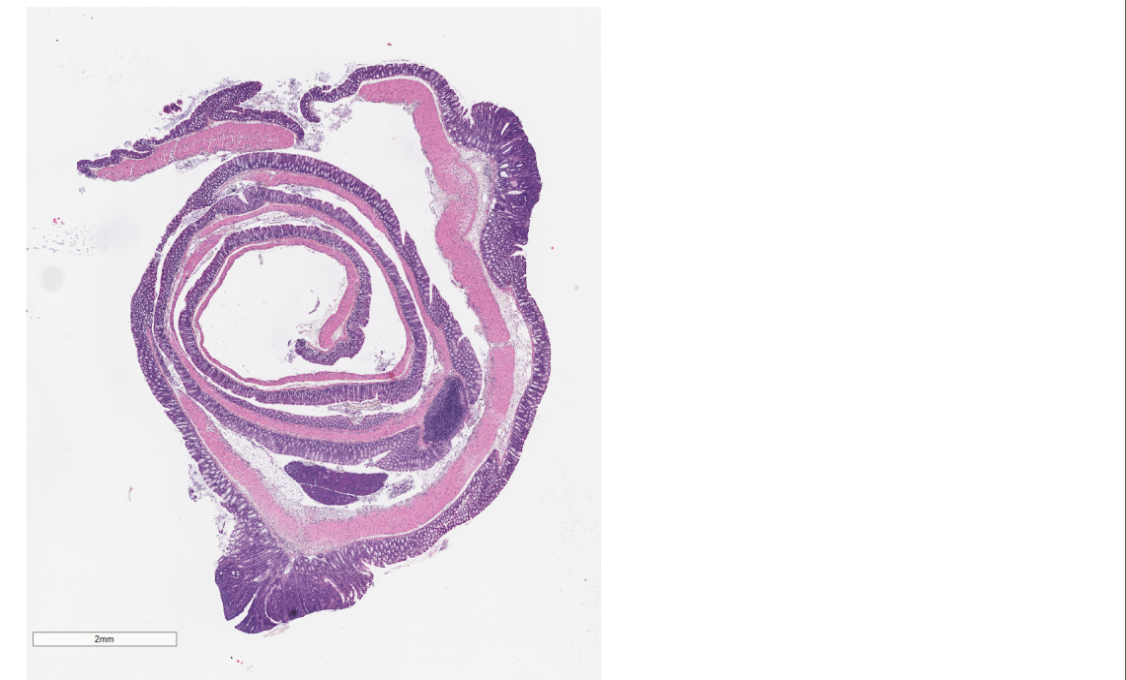

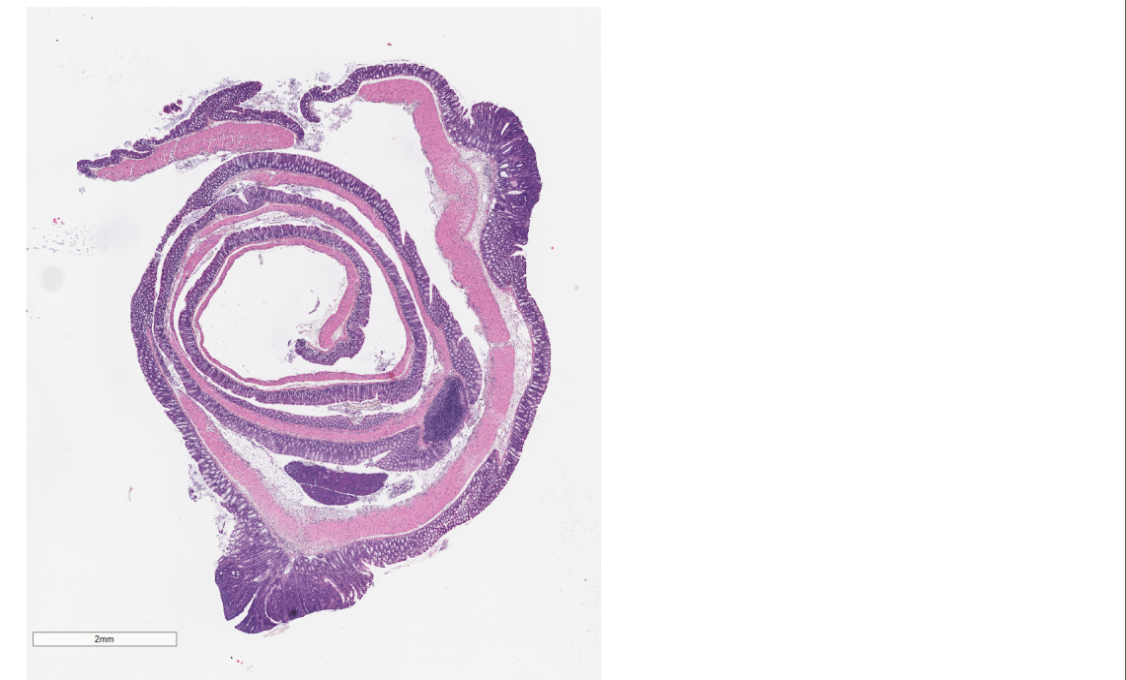


**a**

Tumor

Mucosa

Lymphoid follicle

CD45 analysis, we simply added an export class to


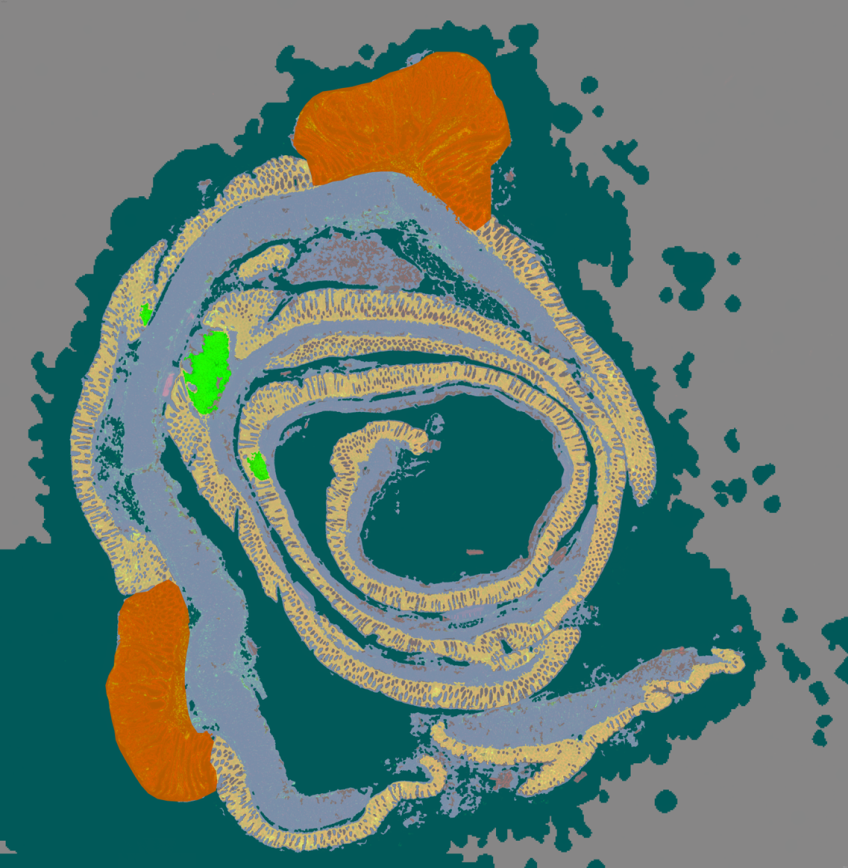

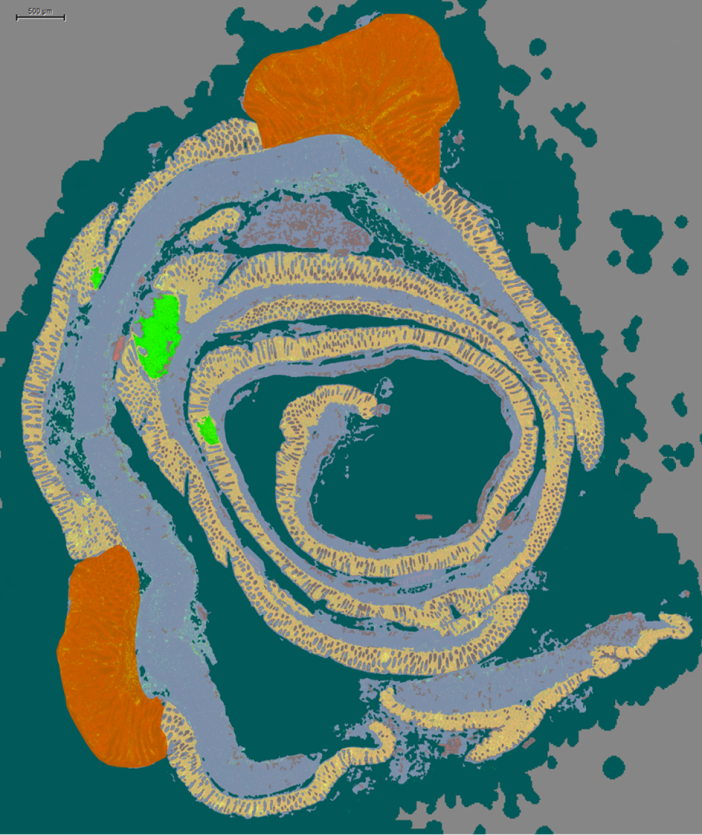


**Tumor**

**b**

Other tissues

Background

No detection

**Mucosa**

**Lymphoid follicle**

**Supplementary figure S3.** A automated method to quantify immune cells on various colon structures. We develop an algorithm to discriminate different colon regions (ROI) on the whole colon (a: HES staining slide; b: result of the algorithm process). (b) Then, we implement cell quantification in three regions of interest that contained immune cells (iROIs): mucosa (yellow), tumors (orange), lymphoid follicles (green).

Use condition :

If Circularity <= 0.0002

**Composer Reclassification Region**

( = Automatic reclassification of fragmented ROI by conditional rules)

Source class :

Tumor

**Composer 1**

Target class :

Mucosa

“ If Tumor object

Circularity Shape Index ≤ 0.0002 ;

Reclassify Tumor object in Mucosa ROI”

**Translation in a conditional rule**

**Algorithm structure created by user in Tissue Studio Software**

Source class :

Tumor

**Composer 2**

Target class :

Mucosa

Use condition :

Area< 300,000

Source class :

Other tissue 2

**Composer 3**

Target class :

Tumor

Use condition :

Relative border to tumor >= 0.3

Source class :

Other tissue

**Composer 4**

Target class :

Tumor

Use condition :

Relative border to tumor >= 0.6

“If Tumor Object Area < 300,000 ;

Reclassify Tumor object in Mucosa ROI”

Source class :

Mucosa

**Composer 5**

Target class :

Tumor

Use condition :

Relative border to tumor >= 0.3

**Composer 6**

Target class :

Lymphoid follicle

Use condition :

Relative border to

Lymphoide follicle >= 0.4

Source class :

Mucosa

**Composer 7**

Target class :

Mucosa

Use condition :

Area <= 10,000

Source class :

Lymphoide follicle

**Composer 8**

Target class :

Other tissue 2

Use condition :

Area <= 10,000

Source class :

Mucosa

“If Relative border of Other tissue 2 object

to Tumor object ≥ 0.3 ;

Reclassify Other tissue 2 object in Tumor ROI”

“If Relative border of Other tissue 1 object

to Tumor object ≥ 0.6 ;

Reclassify Other tissue object in Tumor ROI”

“If Relative border of Mucosa object

to Tumor object ≥ 0.3 ;

Reclassify Mucosa object in Tumor ROI”

“If Relative border of Mucosa object

to Lymphoide follicle object ≥ 0.4 ;

Reclassify Mucosa object in Lymphoide follicle ROI”

“If Lymphoide follicle area ≤ 10,000;

Reclassify lymphoide follicle object in Mucosa ROI”

“If Mucosa area ≤ 10,000;

Reclassify Mucosa object in Other tissue 2 ROI”

**Supplementary figure S4.** ROI reclassification rules details for CD3CD4 double staining. In the same way that CD45 simple staining, we use a ROI reclassification step to improve the ROI classification step precision. Same features are used to implement the conditional rules: Area of object (**Composer 2**, **7** and **8**) and the proximity between two objects (“relative border to”; **Composer 3**-**6**). We add a new feature to improve this reclassification based on three different fluorescence markers by implementing conditional rule based on the object shape and more precisely on the object circularity (**Composer 1**). Composer: ruleset.

| **Coded available action set** | **Block Action Goal** (linked with whole colon slide analysis) | **List of available coded actions (for each block action)** | **Available function** | **Function-associated parameters** | **Total number of possible rulesets** |
| --- | --- | --- | --- | --- | --- |
| General Setting | Set the workflow parameter for digital image analysis | Unique fixed solution depend on image acquisition parameters | | - Magnification  - Pixel Size  - Bit Depth  - Fluorescence channel | 1 |
| Tissue detection | Complete detection of whole colon tissue | Unique tissue detection | * Automatic detection | - Tissue minimal size  - Fluorescence Brightness  - Fluorescence Homogeneity | $\approx$6.3 x 10^7^ |
|  |  |  | * Manual detection | NA | NA |
| ROI classification | Distinction of the APC^Min/+^ colon ROIs  ROI Obtained size | - Manual ROI selection | ND | ND | ND |
|  |  | - Initialization composer | * Optimized ROI analysis magnification set | Magnification | 100 |
|  |  | - Training composer | * Selection of sample to allow training based –based discrimination of ROI and automatic ROI classification | - Segmentation scale  - Panel of selected sample | Up to 8 |
|  |  | - Cut a bottleneck composer  - Create a Region composer  - Create Margin | ND | ND | ND |
|  |  | - ROI reclassification | * Reclassify one ROI class to another ROI class based on 3 conditions (= on up to 3 parameters) | 15 parameters for each couple of ROI :  - Fluorescence intensity : mean, standard deviation, pixel ratio between 2 marker  - ROI Geometry : circularity , compactness,  border index, density, elliptic fit, roundness, shape index  -Relation between object : Number of objects, border to object, relative border to an object, area ratio between two objects | $\approx$9.1 x 10^415^  (for analysis of 3 ROIs) |
|  |  | - ROI correction | * Select object  OR/AND  * Select polygon  To correct ROI | NA | 1 |
| Cellular analysis | Detect cells  Classify cells  Quantify cells | -Initialize cell analysis | * Optimized cellular analysis magnification set | Magnification | 6 |
|  |  |  | * Selection of ROI to make cellular analysis | ROI discrimination | Up to 8 |
|  |  | - Nucleus detection | * Detection of nuclei based on nuclear marker | - Fluorescence intensity of nuclear marker  - Typical nuclei size | 18 for a simple staining  or  336 for a double staining |
|  |  | -Nucleus Morphology filter  -Marker area detection  -Vessel detection  - Membranes and cells  - Spot detection  - Marker area classification  - Nucleus classification  - Membrane classification  - Vessel classification | ND | ND |  |
|  |  | - Cell simulation | * Detect cells | - Choice of simulation mode : “inside cytoplasmic stain” or “growth from nuclei”  - Fluorescence intensity of membrane marker |  |
|  |  | - Cell classification | * Classify and quantify cells | - Optimal threshold to distinct positive cells to negative cells, for a marker |  |
| Export Settings | Set parameter to export numeric data | Default export | * Export Numeric data per object  *Export data per ROI object | NA | 3 |
|  |  | Custom export for cellular analysis | * Export all data associated with cellular analysis | 50 parameters based on fluorescence, geometry, position… of cells | ND |
|  |  | Custom export for ROI analysis | * Export all data for ROI analysis | See ROI reclassification parameters | ND |
|  |  | Heat Map | ND | ND | ND |

**Supplementary Table 1.** Overview of available coded actions on Tissue Studio software. Definiens Tissue Studio software provides an exhaustive list of pre-coded functions. These functions are associated with five principal action family (or sets): “General setting”, to set analysis workflow parameters; “Tissue detection”; “ROI classification”, “Cellular Analysis” and “Export settings”. Each set of actions contains different possible coded actions. Besides, various function and parameters could be selected to implement a coded action. It results in a great number of available possible ruleset combinations. This table developed more precisely the actions that we used to develop algorithm to quantify cell on whole APC^Min/+^ mice colon. Thanks to its user-friendly interface, any user can choose and order these functions to build new rulesets, adapted to his/her scientific problematic.

**Supplementary methods**

**Numbering of possible rulesets available in** Tissue Studio **Software.** To determine the number of possible rulesets for each action we calculate the total number of possible combinations. When parameters are fixed by image acquisition we considered that there is one unique possibility, as for “General Settings”. When action contains only one parameter setting as “ROI Classification _Initialization and Training” composer; and “Cellular Analysis_Initialize cell analysis” we simply report the number of possible choice in the software.

For tissue detection, this step is composed of the setting choices. Each marker has the same independent choice of settings. Even if they are used at the same time, we considered that these parameters are independent so the number of combination is calculated by equation (1).

For ROI classification, with the software we can choose to implement up to 11 reclassification rules with a specific order, so there are $\sum_{k=1}^{11} k !$ possibilities of reclassification conditions, with *k* is the chosen number of reclassification rules. For each of these rules we have to choose 1 to 3 conditions, so there are $\sum_{i=1}^{3} \binom{3}{i}$ possibilities of condition choices, with *i* is the chosen number of the conditions. Finally, for each condition we have to choose 1 to 40 parameters, so there are $\sum_{j=1}^{40} \binom{40}{j}$ possibilities of parameters choices, with *j* is the number of chosen parameters. For these conditional rules we have to choose 2 of 5 ROIs defined in the algorithm, so there are $\binom{5}{2}$ of ROIs couple. In conclusion the number of ROI reclassification succession rules is calculated by equation (2).

For cellular analysis, if we are interested only in nuclei and membrane object, there are 2 possibilities to detect nuclei and 3 possibilities to detect membrane. Then, to classify cells we can choose $\binom{3}{2}$ condition of marker expression or co-expression for a simple staining for example. So the number of Cellular analysis rules is calculated by the equation (3).

**Supplementary equations**

1. **N _marker 1 (Tissue Min Size)_ x N _marker 2 (Fluorescence Brightness) x_ N _marker 3 (Fluorescence Homogeneity)_**

= 51 x 255 x 301

$\approx$ 3.9 x 10^6^ possible rules combinations

With N: number of possibility for a parameter

1. $\sum_{\boldsymbol{k=1}}^{\boldsymbol{11}} \boldsymbol{k ! \times}{{\boldsymbol{((}\binom{\boldsymbol{5}}{\boldsymbol{2}}\boldsymbol{\times}\sum_{\boldsymbol{i=1}}^{\boldsymbol{3}} \binom{\boldsymbol{3}}{\boldsymbol{i}}\boldsymbol{\times}\sum_{\boldsymbol{j=1}}^{\boldsymbol{40}} \binom{\boldsymbol{40}}{\boldsymbol{j}}\boldsymbol{)}}^{\boldsymbol{i}}\boldsymbol{)}}^{\boldsymbol{k}}$

$\approx$ 9.1 x 10^415^ possible rules combinations

1. **2 x 3 x** $\binom{\boldsymbol{3}}{\boldsymbol{2}}$

= 18 possible rules for cellular analysis of a simple staining
